# A Gene Feature Based on Histone Modifications Can Predict the Prognosis of Prostate Cancer

**DOI:** 10.3390/biomedicines14061219

**Published:** 2026-05-28

**Authors:** Jialin Gao, Xuee Zhou, Zetao Zuo, Jiahong Hong, Yan Tan, Xiaoxiang Rong, Rui Zhou, Zhenhua Huang

**Affiliations:** Department of Oncology, Nanfang Hospital, Southern Medical University, Guangzhou 510515, China; gaotutu0929@163.com (J.G.); xezhou21@163.com (X.Z.); wdmzszzt@163.com (Z.Z.); hongjh3@163.com (J.H.); 13535340435@163.com (Y.T.)

**Keywords:** histone modification, prognosis, prediction model, prostate cancer, nomogram

## Abstract

**Background/Objectives**: Prostate cancer (PCa) remains a prevalent malignancy among men, often complicated by recurrence and unfavorable clinical outcomes. Consequently, precise risk stratification and timely clinical intervention are paramount. Initially, we delineated distinct expression profiles of histone modification regulators via unsupervised clustering, identifying PCa subtypes with divergent survival probabilities and biological phenotypes. Subsequently, we sought to develop a prognostic gene signature, derived from the transcriptomic variations among these regulator-defined subtypes, to predict outcomes in PCa patients following radical prostatectomy (RP). **Methods**: Clinical and transcriptomic data from PCa cohorts were retrieved from The Cancer Genome Atlas (TCGA) and Gene Expression Omnibus (GEO) repositories for comprehensive analysis. Subtypes driven by histone modification regulators were established using unsupervised consensus clustering, followed by in-depth characterization of their molecular features and associated pathways. A risk-scoring model was then developed to evaluate its prognostic efficacy in this patient population. **Results**: Stratification based on histone modification regulators yielded four distinct PCa subtypes exhibiting heterogeneous survival outcomes, functional pathways, and genomic mutational landscapes. Following rigorous feature selection, a 21-gene risk signature (HIS_score)—comprising MXD3, CCDC28B, COL11A2, SLC39A5, GPT, DNASE1L2, PIF1, KRTAP5-9, TTLL10, KRTAP5-1, KRTAP5-10, HAGHL, MSLNL, AMH, NKAIN4, CCDC114, SLC9A3, SULT1E1, ALB, SLC6A14, and RPE65—was constructed. Survival analyses demonstrated that patients assigned to the high HIS_score cohort experienced significantly worse clinical outcomes compared to their low-score counterparts. Furthermore, we integrated this signature into a novel clinical nomogram to facilitate individualized prognostic assessments. **Conclusions**: Derived from transcriptomic disparities between extreme epigenetic subtypes, the HIS_score and its associated nomogram serve as robust prognostic instruments. These tools effectively encapsulate the downstream transcriptional sequelae of histone modification dysregulation, offering clinicians a valuable framework to accurately predict post-RP outcomes and expedite the formulation of personalized therapeutic strategies.

## 1. Introduction

Prostate cancer (PCa) is a highly heterogeneous disease and a prevalent malignancy among men worldwide. It is also a major cause of cancer-related deaths in men [1]. Curative-intent management for organ-confined, androgen-dependent PCa includes modalities such as radical prostatectomy (RP), radiotherapy, and androgen deprivation therapy [2]. Despite the clinical efficacy of these therapeutic modalities, approximately one-third of patients experience disease recurrence and progression following RP. This trajectory frequently culminates in the development of castration-resistant PCa, which typically leads to mortality within 2–4 years [3,4,5]. Consequently, the precise identification of high-risk patients prone to postoperative recurrence or progression is imperative for the formulation of tailored therapeutic interventions. Historically, clinicopathological parameters, including the Gleason score, prostate-specific antigen (PSA) levels, and clinical T stage, have been utilized to estimate patient survival and predict post-RP prognosis [6]. Nevertheless, the inherent limitations of these conventional metrics often constrain their predictive accuracy.

In recent years, researchers have conducted a series of studies to better identify patients with poor prognosis and explored gene signatures based on transcriptomic and clinical data to predict PCa outcomes. For example, Lv et al. established and validated a seven-gene signature based on immune-related genes to monitor immune status and evaluate recurrence-free survival (RFS) rate of PCa patients [7]. Similarly, Zhang et al. identified an apoptosis-related gene signature and improved the risk stratification of RFS by integrating this signature with clinical parameters, thus identifying patients with a poor prognosis more accurately [8].

Epigenetic reprogramming plays a pivotal role in driving the progression of PCa [9,10]. Epigenetic regulation, which is primarily mediated through DNA methylation, histone modifications, and microRNA expression, is fundamental to cancer pathogenesis [11]. Among these mechanisms, histone modifications serve as critical, highly complex epigenetic coordinators of chromatin architecture, DNA damage repair, and transcriptional regulation. Accumulating evidence suggests that aberrant histone modifications are intimately linked to PCa initiation and progression, profoundly influencing key cellular processes, including proliferation, apoptosis, and metastatic dissemination [12]. Research has shown that global changes in histone modifications in cancer cells can predict the risk of tumor recurrence in low-grade PCa patients independently of tumor stage, preoperative PSA, and capsular invasion [13]. To date, however, the practical application of histone modification profiles in PCa diagnostics and therapeutics remains underexplored [14]. There has been no exploration into whether the global transcriptional expression levels of histone-modifying factors can distinguish high-risk PCa patients, indicating that substantial opportunities remain for the development and application of histone modifications in PCa.

To address this, our study first applied unsupervised consensus clustering to the transcriptomic profiles of 67 rigorously curated histone modification regulators, successfully delineating biologically distinct PCa subtypes with divergent prognostic trajectories. Subsequently, we constructed a robust prognostic gene signature (termed HIS_score) based on the core transcriptomic disparities between the two subtypes exhibiting the most extreme clinical outcomes (C1 versus C3). This regulator-guided framework links upstream epigenetic patterning with downstream transcriptional consequences, ultimately aiming to refine the risk stratification of patients following RP. We anticipate that these findings will facilitate clinical decision-making, offering a novel tool to optimize personalized therapeutic strategies and improve survival outcomes for patients with PCa.

## 2. Materials and Methods

### 2.1. Study Population, Data Acquisition, and Processing

This retrospective study utilized publicly available gene expression and clinical datasets from The Cancer Genome Atlas (TCGA, https://cancergenome.nih.gov/, accessed on 24 May 2026) and the National Center for Biotechnology Information Gene Expression Omnibus (GEO). To specifically evaluate the post-RP clinical setting, two independent cohorts of patients with primary prostate adenocarcinoma (PRAD) who underwent curative-intent RP were included: TCGA-PRAD and GSE70770. Patients within the TCGA-PRAD cohort were selected if their documented surgical procedure was RP, ensuring no prior neoadjuvant interventions (chemotherapy, radiotherapy, or androgen deprivation therapy) had been administered before tissue collection. For the GSE70770 cohort, primary treatment with RP was verified via the accompanying clinical metadata. The inclusion criteria for both datasets comprised: (1) histologically confirmed primary PRAD; (2) RP as the primary surgical intervention; and (3) complete baseline clinical and survival records. Patients were excluded based on: (1) metastatic disease (M1) at initial diagnosis; or (2) missing essential clinicopathological metrics (PSA, Gleason score, or TNM stage) and survival outcomes. Both node-positive and node-negative individuals were eligible for inclusion. Applying these criteria yielded a final analytical cohort of 495 TCGA-PRAD patients and 203 GSE70770 patients, complete with matched clinical data (Supplementary Table S1). Extracted clinical variables encompassed age, TNM stage, PSA levels, and Gleason score. Corresponding genomic mutation profiles for TCGA-PRAD were sourced from the University of California, Santa Cruz (UCSC) Xena database. RNA-seq count data underwent preprocessing via the voom algorithm in the limma R package (version 3.48.0), which models the mean-variance relationship to generate homoscedastic log-counts optimized for linear modeling and differential expression evaluation. Preprocessed and normalized expression matrices for GSE70770 were directly retrieved from GEO. To preserve data integrity, cross-platform batch correction between the TCGA-PRAD RNA-seq and GSE70770 microarray datasets was intentionally omitted. Instead, the HIS_score was calculated independently within each respective cohort, applying the fixed 21-gene coefficients established in TCGA, followed by stratification based on the cohort-specific median score (detailed in Section 2.3). This internal ranking approach effectively circumvents potential platform-specific systematic biases.

Within the TCGA-PRAD cohort, the primary endpoint was the progression-free interval (PFI), defined as the duration from surgical intervention to biochemical recurrence (BCR), local/distant progression, or all-cause mortality. The disease-free interval (DFI) served as a secondary endpoint strictly for the subset of patients achieving R0 resection. For the independent validation cohort (GSE70770), disease-free survival (DFS)—measured from surgery to BCR or death—was assigned as the primary endpoint. Despite minor variations in event definitions across these datasets (primarily concerning the exact capture of biochemical versus clinical progression), PFI, DFI, and DFS remain conceptually unified as recurrence- or progression-free survival metrics specific to the post-RP context. While these composite endpoints encompass diverse progression modes, it should be explicitly noted that BCR constitutes the predominant event driving the survival curves. This distribution aligns with standard clinical observations in post-RP cohorts, where BCR typically emerges as the earliest and most frequent manifestation of disease relapse. Consequently, all survival analyses utilized these cohort-specific endpoints to maximize data fidelity, thereby validating the consistent prognostic capability of the HIS_score across diverse clinical measures. Notably, in Supplementary Figure S2, an “Event” strictly denotes the incidence of BCR, clinical progression (local or metastatic), or death during the follow-up period.

### 2.2. Identification of Histone Modification Regulator Expression Patterns by Consensus Clustering

Unsupervised K-means clustering was applied to delineate distinct histone modification regulator expression patterns and subsequently stratify PCa patients. To evaluate clustering stability and ascertain the optimal number of clusters (k), we implemented the consensus clustering algorithm using the “ConsensusClusterPlus” R package [15]. Algorithm parameters were configured as follows: 80% item resampling (pItem = 0.8), 80% feature resampling (pFeature = 0.8), a maximum k of 10 (maxK = 10), 1000 iterations (reps = 1000), and the K-means algorithm utilizing the Euclidean distance metric (clusterAlg = “km”, distance = “euclidean”).

### 2.3. Generation and Validation of a Histone Modification Regulator-Guided Prognostic Model

To develop a risk-scoring model capturing the downstream transcriptomic consequences of histone modification regulator dysregulation, we initially conducted unsupervised consensus clustering across the entire TCGA-PRAD cohort (*n* = 495) to isolate biologically distinct subtypes. This exploratory approach identified two subgroups exhibiting highly divergent clinical trajectories. We specifically selected these extreme clusters—C1 (favorable prognosis) and C3 (poor prognosis)—because they anchor opposite ends of the epigenetic prognostic spectrum. This strategy maximizes the detection of transcriptomic alterations associated with disease progression while filtering out the noise inherent to intermediate phenotypes. Differentially expressed genes (DEGs) discriminating C1 from C3 were identified using the limma R package [16], applying thresholds of an adjusted *p*-value < 0.05 and |log2FC| > 1. To rigorously mitigate data leakage and the risk of overfitting, these candidate genes were subjected to univariate Cox regression and Kaplan–Meier survival analyses, followed by a penalized least absolute shrinkage and selection operator (LASSO) regression (1000 iterations, 10-fold cross-validation) strictly within the TCGA-PRAD training set (*n* = 495). This pipeline ultimately yielded the 21-gene HIS_score signature. The HIS_score was calculated by subtracting the average expression level of the 2 protective genes from the average expression level of the 19 risk-associated genes (the detailed formula and the complete list of 21 genes with their risk/protective roles are provided in Supplementary Table S6). Within the TCGA-PRAD training cohort, patients were dichotomized into high- and low-risk groups based on the median HIS_score threshold. Kaplan–Meier survival analyses were subsequently executed to evaluate prognostic discrepancies between these risk strata. To validate prognostic robustness, this identical scoring algorithm was independently applied to the GSE70770 validation cohort (*n* = 203). While fundamentally data-driven, our methodological integration of biology-guided feature prioritization, rigorous internal cross-validation, and external validation inherently mitigates the risks of overfitting and selection bias. To circumvent potential batch effects confounding RNA-seq and microarray comparisons, the HIS_score was computed independently within each dataset using the pre-established scoring logic. Samples were then re-stratified into high- and low-risk categories contingent on the cohort-specific median. By relying on relative intra-cohort expression distributions rather than absolute cross-platform quantifications, this strategy systematically minimizes technical bias and reinforces the external validity of the 21-gene signature. Finally, the prognostic efficacy of the HIS_score was assessed using PFI as the primary endpoint for TCGA-PRAD, and DFS for GSE70770. This approach accommodates dataset-specific survival definitions while preserving overall analytical integrity.

### 2.4. CNV Mutation Profile and Methylation Characteristics

To systematically evaluate genomic alterations, somatic copy number variation (CNV) profiles were retrieved using the “TCGAbiolinks” R package [17]. Concurrently, corresponding 450K DNA methylation array data for the TCGA-PRAD dataset were sourced from the UCSC Xena database (https://xenabrowser.net). A landscape analysis of focal CNVs, encompassing both deletion and amplification frequencies across the entire cohort, was conducted using the GISTIC 2.0 algorithm. For epigenetic profiling, a stringent probe-filtering pipeline was implemented: probes located on sex chromosomes and those containing single-nucleotide polymorphisms (SNPs) were systematically excluded. Focus was specifically directed toward CpG sites mapped to classical promoter regulatory elements, defined as the “TSS1500”, “TSS200”, “1stExon”, and “5′UTR” regions. Following this filtering step, the 1000 most highly variable probes were subjected to differential methylation analysis via the “ChAMP” R package.

### 2.5. Analysis of Biological Processes and Tumor Microenvironment Features

We evaluated subtype-specific pathway variations using the “GSVA” R package [18], relying on the Hallmark reference sets curated from the Molecular Signatures Database (MSigDB). To map the immune microenvironment landscape, we applied the CIBERSORT algorithm [19] to our gene expression matrices. This deconvolution was executed using the LM22 leukocyte signature matrix, running 1000 permutations with quantile normalization enabled.

### 2.6. Prediction of Immunotherapy and Chemotherapy Response

To predict the clinical responses of PCa patients to immunotherapy, we employed the Tumor Immune Dysfunction and Exclusion (TIDE) algorithm [20]. Furthermore, to assess chemotherapy sensitivity, we estimated the half-maximal inhibitory concentration (IC50) values for 10 commonly used drugs [21] within the TCGA-PRAD cohort. This was achieved using the pRRophetic R package, based on data from the Genomics of Drug Sensitivity in Cancer (GDSC, https://www.cancerrxgene.org) database.

### 2.7. Statistical Analysis

All statistical analyses were performed using R software (version 4.0.5; R Foundation for Statistical Computing, Vienna, Austria) and IBM SPSS Statistics (version 26.0; IBM Corp., Armonk, NY, USA). For all figure annotations, “ns” indicates non-significant, * *p* < 0.05, ** *p* < 0.01, *** *p* < 0.001, and **** *p* < 0.0001. Unless otherwise specified, all statistical tests were two-sided, and a *p*-value < 0.05 was considered statistically significant. To evaluate survival differences across subtypes, the survival R package was employed to generate Kaplan–Meier survival curves, and the log-rank test was used to compare differences between groups. Receiver operating characteristic (ROC) curves were generated to validate the predictive performance of the model, and the area under the curve (AUC) was calculated. Decision curve analysis (DCA) [22,23] was conducted to evaluate the clinical utility of each indicator. Other statistical methods used in this study included the Kruskal–Wallis test, one-way ANOVA, Cox regression analysis, and Pearson correlation analysis.

## 3. Results

### 3.1. Classification and Characteristics of Histone Modification Regulatory Factors in PCa

**Figure 1:**
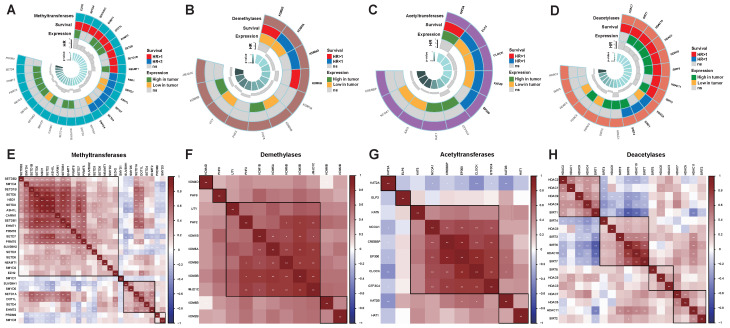
Characterization of histone modification regulators. (**A**–**D**) Circos plots displaying the relationships between histone modification regulators—specifically methyltransferases (**A**), demethylases (**B**), acetyltransferases (**C**), and deacetylases (**D**)—and the samples in the TCGA-PRAD cohort. Progressing from the outermost to the innermost layer, the first ring details the histone modification regulators; black font indicates genes affecting patient prognosis, whereas gray font denotes those without prognostic impact. The second ring illustrates the correlation between these regulatory factors and patient survival. Red indicates genes with an HR greater than 1, blue indicates an HR less than 1, and gray denotes a lack of statistical significance. The third ring (Expression) profiles the expression levels of these genes within the TCGA-PRAD cohort. Green represents higher expression in tumor tissues relative to normal tissues, yellow represents lower expression, and gray indicates no statistical significance. The next gray ring represents the HR value distribution for each factor, with the baseline set at HR = 1. The innermost ring maps the statistical significance of the survival differences, with darker shades corresponding to lower *p*-values. (**E**–**H**) Correlation heatmaps for the histone modification regulators, including methyltransferases (**E**), demethylases (**F**), acetyltransferases (**G**), and deacetylases (**H**). Negative and positive correlations are highlighted in blue and red, respectively.

Following a systematic review of the literature on epigenetic modulation, we curated a comprehensive panel of 67 core histone modification regulatory genes [24,25,26,27,28]. This selection was predicated upon their validated enzymatic roles as writers, readers, or erasers of histone marks, alongside their documented involvement in the epigenetic landscape of oncogenesis, with a specific focus on PCa. Inclusion in this panel required meeting the following strict criteria: (1) encoding an enzyme with experimentally verified histone acetyltransferase, deacetylase, methyltransferase, or demethylase activity; (2) demonstrating functional validation across at least two independent studies linking the gene to tumor progression, transcriptional regulation, or patient prognosis; and (3) excluding targets reliant exclusively on computational predictions lacking empirical validation. This rigorous filtering strategy yielded 28 histone acetyltransferases, 11 histone deacetylases, 10 histone methyltransferases, and 18 histone demethylases (Supplementary Table S2). By employing this literature-driven, family-stratified methodology, we ensured the robustness and breadth of the gene signature, thereby facilitating a systematic evaluation of global transcriptional profiles for histone modifiers across the PCa patient cohort. We evaluated the clinical significance of these regulatory elements by acquiring transcriptome profiles for 495 PCa samples alongside 52 non-cancerous tissues from the TCGA database (detailed in Supplementary Table S3). Further investigations within the TCGA-PRAD dataset demonstrated heterogeneity in both the expression landscapes and the prognostic value of these specific genes. Notably, the expression levels of histone modification regulators differed significantly between PCa and normal tissues. Tumor tissues exhibited an upregulation of 28 genes, largely dominated by methyltransferases (46.43%) and deacetylases (35.71%), whereas exactly two-thirds (66.67%) of the demethylases showed suppressed expression. Survival analysis of the PFI across the 67 regulators indicated that high expression of several histone modifiers predicted worse survival. Deacetylases constituted the majority (60%) of these prognostic risk factors, marking the highest fraction among the four enzyme types (Figure 1A–D, Supplementary Table S4). Furthermore, co-expression analyses uncovered largely positive regulatory networks within individual families (Figure 1E–H), with acetyltransferases exhibiting the tightest intra-family associations. Moreover, an inverse relationship was noted between the methyltransferase cluster and KAT2A, a gene previously validated as a PCa-specific transcriptional activator by Simmonds et al. [29]. Overall, these findings indicate that histone modification regulators exert critical effects on the development and prognosis of PCa. To augment the visual data provided by the circos plots (Figure 1) and offer a concise overview of critical clinical metrics, Table 1 details all histone-modifying genes significantly linked to a shorter PFI (HR > 1, *p* < 0.05). The prominence of methyltransferases and deacetylases among these poor-prognosis markers highlights their potential as highly relevant targets for future mechanistic and therapeutic investigations.

### 3.2. Construction of Histone Modifier Expression Pattern Subtypes for PCa

**Figure 2:**
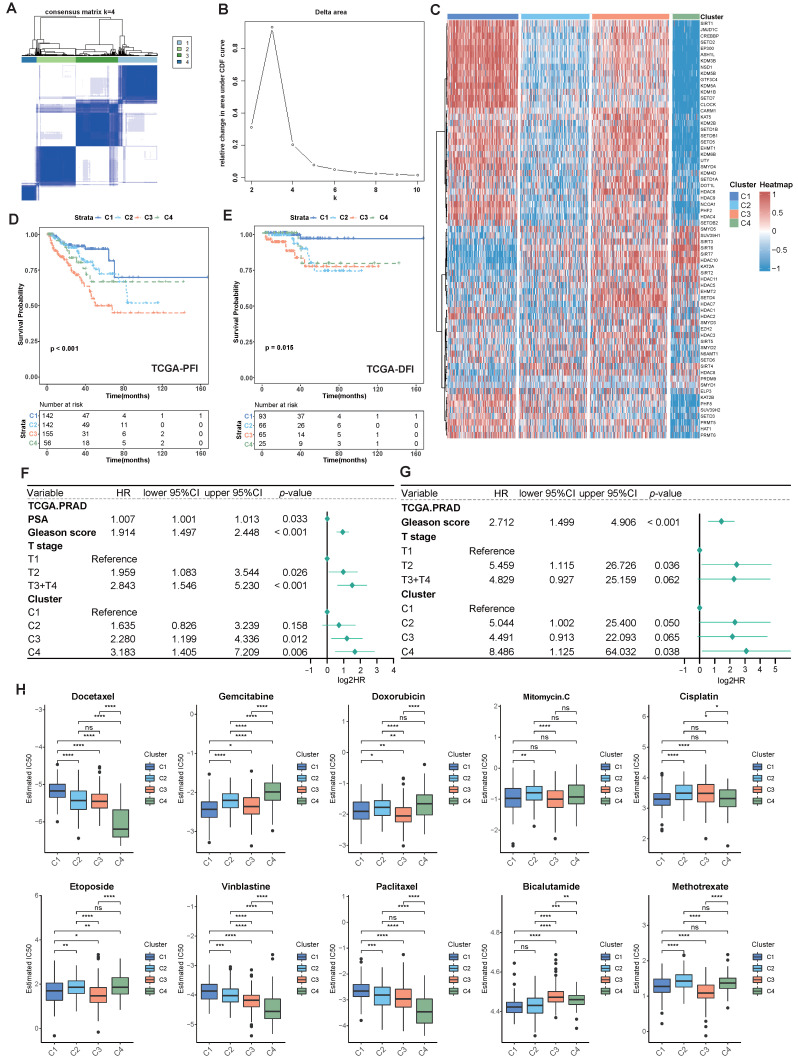
Identification of four PRAD clusters based on histone modification regulators. (**A**) Consensus clustering matrix for k = 4. The range of consensus values is from 0 to 1, with 0 indicating never clustered (white) and 1 indicating always clustered (dark blue). (**B**) Relative change in area under the cumulative distribution function (CDF) curve for different values of k. (**C**) Heatmap depicting the expression profiles of 67 histone modification regulators in 495 patients from the TCGA-PRAD cohort, stratified into four consensus clusters. Clusters are color-coded as follows: dark blue (C1), sky blue (C2), orange (C3), and green (C4). (**D**) Kaplan–Meier survival curves of PFI for the four regulator-defined PRAD clusters based on 495 patients from the TCGA-PRAD cohort. (**E**) Kaplan–Meier survival curves of DFI for the four regulator-defined PRAD clusters based on 249 patients from the TCGA-PRAD cohort. (**F**,**G**) Forest plots showing the effects of the four clusters and clinical characteristics on PFI (**F**) and DFI (**G**) in a multivariable analysis, which included PSA, Gleason score, T stage, and the four PRAD clusters. The length of the horizontal line represents the 95% confidence interval (CI). (**H**) Estimated IC50 values for 10 common chemotherapeutic agents across the four clusters. Statistical differences among groups were assessed using the Kruskal–Wallis test. *: *p* < 0.05, **: *p* < 0.01, ***: *p* < 0.001, ****: *p* < 0.0001; ns: not significant (*p* > 0.05).

We investigated whether transcriptomic profiling of the 67 aforementioned histone modification regulators could facilitate the stratification of PCa patients. Consequently, their gene expression profiles were utilized to delineate molecular subtypes. The optimal number of clusters (k), evaluated from 2 to 10, was determined via a consensus clustering algorithm. Stable clustering was achieved at k = 4 (Figure 2A,B), successfully stratifying PCa into four distinct subtypes based on their histone modification regulator expression patterns (designated as PRAD clusters): C1 (*n* = 142), C2 (*n* = 142), C3 (*n* = 155), and C4 (*n* = 56). These clusters exhibited notable transcriptomic heterogeneity; specifically, C3 patients demonstrated elevated expression levels across nearly all histone modification regulators, implying heightened epigenetic activity, whereas the remaining subtypes displayed only partial enrichment of these factors (Figure 2C). Survival analyses revealed profound disparities in both PFI and DFI across these regulator-defined subgroups. Regarding PFI, C3 patients experienced significantly worse clinical outcomes compared to the C1, C2, and C4 cohorts, with C1 displaying the most favorable prognosis (*p* < 0.001, Figure 2D). The 3-year PFI rates for the respective PRAD clusters were 92.96%, 88.03%, 76.77%, and 85.71%. Similarly, DFI analysis restricted to the 249 patients undergoing R0 resection confirmed that the C1 group retained the most favorable outcome (5-year DFI rate of 97.32%), whereas C3 patients were at an elevated risk of recurrence relative to the other subtypes (*p* = 0.015, Figure 2E). Subsequent univariate Cox regression incorporating age, PSA, Gleason score, clinical T stage, and the PRAD clusters identified several clinically significant variables (Supplementary Figure S1A,B). Furthermore, multivariate Cox analysis revealed that these regulator-defined PRAD clusters served as independent prognostic determinants for PFI within the TCGA-PRAD cohort. Specifically, compared to C1, the C4 subtype was associated with a substantially increased risk of both progression (*p* = 0.006, hazard ratio [HR] = 3.183, 95% confidence interval [CI] = 1.405–7.209, Figure 2F) and relapse (*p* = 0.038, HR = 8.486, 95% CI = 1.125–64.032, Figure 2G). The C3 subtype similarly presented an elevated progression risk (*p* = 0.012, HR = 2.280, 95% CI = 1.199–4.336, Figure 2F). Finally, to evaluate differential chemotherapeutic sensitivities, we estimated the IC50 for 10 conventional anti-PCa agents across the four subtypes. The analysis indicated that C1 tumors exhibited greater sensitivity to gemcitabine, cisplatin, and bicalutamide compared to the other clusters (Figure 2H). Within the poor-prognosis cohorts (C3 and C4), the C4 subtype displayed the lowest IC50 values for docetaxel, vinblastine, and paclitaxel. Conversely, C3 tumors demonstrated enhanced susceptibility to gemcitabine, doxorubicin, mitomycin C, etoposide, and methotrexate when compared to C4. Further predictive modeling of additional chemotherapeutics confirmed widespread, subtype-specific variances in drug sensitivity (Supplementary Figure S1C). Collectively, these findings suggest that divergent expression patterns of histone modification regulators dictate distinct prognostic trajectories and hold profound implications for personalized therapeutic strategies. These pronounced clinical and pharmacological disparities subsequently prompted a deeper investigation into the underlying biological pathways and immune microenvironmental landscapes distinguishing these regulator-defined subtypes.

### 3.3. Biological Functions and Tumor Microenvironment Immune Cell Infiltration of Histone Modification Regulator Expression Pattern Subtypes

**Figure 3:**
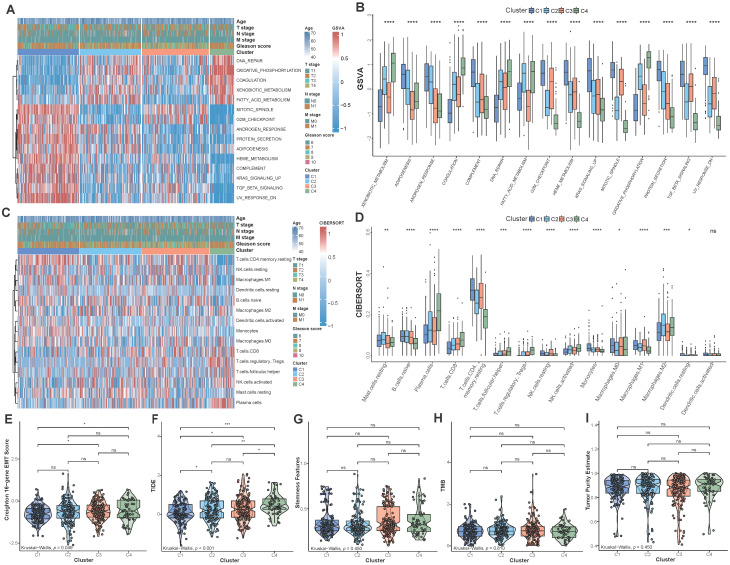
Biological and tumor microenvironment profiles across distinct PRAD clusters. (**A**) Heatmap illustrating the GSVA enrichment scores of representative hallmark pathways across the four PRAD clusters. Red indicates pathway activation, and blue indicates pathway suppression. PRAD clusters and clinical covariates (Age, TNM stage, and Gleason score) are annotated for each sample. (**B**) Box plots depicting the GSVA scores for hallmark pathways across the four PRAD clusters in the TCGA-PRAD cohort. The boxes encompass the 25th to 75th percentiles; the horizontal line within each box indicates the median, the whiskers extend to 1.5 times the interquartile range, and the black dots represent outliers. (**C**) Infiltration profiles of tumor microenvironment-infiltrating cells stratified by the different histone modification regulator expression patterns. PRAD clusters and clinical covariates of the TCGA-PRAD cohort (age, TNM stage, and Gleason score) are annotated for each sample. (**D**) Proportions of tumor microenvironment-infiltrating cells, estimated using the CIBERSORT algorithm, across the four PRAD clusters. The boxes encompass the 25th to 75th percentiles; the horizontal line within each box indicates the median, the whiskers extend to 1.5 times the interquartile range, and the black dots represent outliers. (**E**–**I**) Distributions of EMT scores (**E**), TIDE scores (**F**), stemness scores (**G**), TMB scores (**H**), and tumor purity (**I**) across the four PRAD clusters from the TCGA-PRAD cohort. Statistical differences among the groups were evaluated using the Kruskal–Wallis test. *: *p* < 0.05, **: *p* < 0.01, ***: *p* < 0.001, ****: *p* < 0.0001; ns: not significant (*p* > 0.05).

Molecular phenotyping via Gene Set Variation Analysis (GSVA) uncovered stark functional divergence among the defined PRAD classifications (Figure 3A,B). A central feature of this heterogeneity involved distinct metabolic state transitions. Specifically, energetic and biosynthetic networks crucial to prostate tumorigenesis—including oxidative phosphorylation, fatty acid metabolism, and biosynthesis of amino acids—were markedly downregulated in the C1 and C3 cohorts. Conversely, the C2 and C4 subgroups maintained hyperactive metabolic profiles. Beyond these metabolic shifts, the C3 cluster was intrinsically characterized by a severe blunting of the androgen receptor signaling cascade, a pathway that remained functionally robust in the C1 and C2 subtypes. Additionally, an exclusive upregulation of complement-related immune signaling was identified within the C1 group. These variations, particularly in androgen signaling and complement activation, may underlie the divergent clinical outcomes observed among the subtypes. Additionally, C1 patients showed enrichment for several oncogenic cascades, such as the KRAS and TGF-β signaling pathways [30]. Interestingly, cell cycle-related pathways (including the G2M checkpoint and mitotic spindle assembly) were highly activated in both C1 and C3 patients but significantly repressed in C2 and C4 patients (Figure 3A,B). Although both C1 and C3 exhibited heightened cell cycle activation, C1 patients experienced the best prognosis, whereas C3 patients presented with the worst. This apparent paradox may be explained by underlying differences in androgen response (AR) signaling, complement pathway activation, and CNV burden (detailed in Section 3.4). Specifically, C1 tumors showed a relatively preserved AR and strong complement activation coupled with M1 macrophage skewing, which may counteract proliferative signals. In contrast, C3 tumors exhibited stronger AR suppression and a higher CNV burden, potentially leading to greater genomic instability and phenotypic aggressiveness. Regarding the tumor immune microenvironment (Figure 3C,D), a heatmap and box plots were generated to visualize the distribution of 15 distinct tumor-infiltrating immune cell populations. Several immune cell subsets with crucial regulatory and cytotoxic functions, such as plasma cells, CD8+ T cells, T follicular helper cells, regulatory T cells (Tregs), and activated natural killer cells, were enriched in C4 patients. Although CD8+ T cell infiltration was at its highest in the C4 subgroup, the proportion of immunosuppressive Tregs was also significantly elevated compared to the other subtypes. Concurrently, macrophages in C4 patients tended to polarize towards the pro-tumorigenic M2 phenotype. Similar to C4 patients, C2 patients also showed a tendency towards M2 polarization, while C1 and C3 patients exhibited a tendency towards M1 polarization, with the relative proportion of monocytes in C1 patients being significantly higher than in the other subtypes.

Additionally, we calculated the TIDE scores and obtained the distribution of epithelial–mesenchymal transition (EMT) scores [31], stemness characteristics [32], tumor mutational burden (TMB) [32], and tumor purity [33] for the TCGA-PRAD cohort from the available literature. Our analysis showed that C1 patients had the lowest EMT and TIDE scores, suggesting a lower invasive potential and a higher likelihood of benefiting from immune checkpoint blockade. Conversely, C4 patients presented with the highest TIDE score, indicative of a potentially more immunosuppressive microenvironment. Importantly, TIDE scores are inferred from bulk transcriptomic data and do not directly measure T cell exhaustion markers; thus, these interpretations are hypothesis-generating and warrant further experimental validation. There were no significant differences in stemness, TMB, or tumor purity among the subtypes (Figure 3E–I). These results emphasize the existence of four distinct histone modification regulator expression patterns in PCa, which represent diverse biological behaviors and tumor microenvironment profiles that ultimately dictate differences in prognosis. Given the distinct biological and immune landscapes across the regulator-defined subtypes, we next examined whether these classifications also differed at the genomic level, particularly concerning copy number alterations and DNA methylation patterns.

### 3.4. Mutation Profiles and Genetic Alterations Among Subtypes

**Figure 4:**
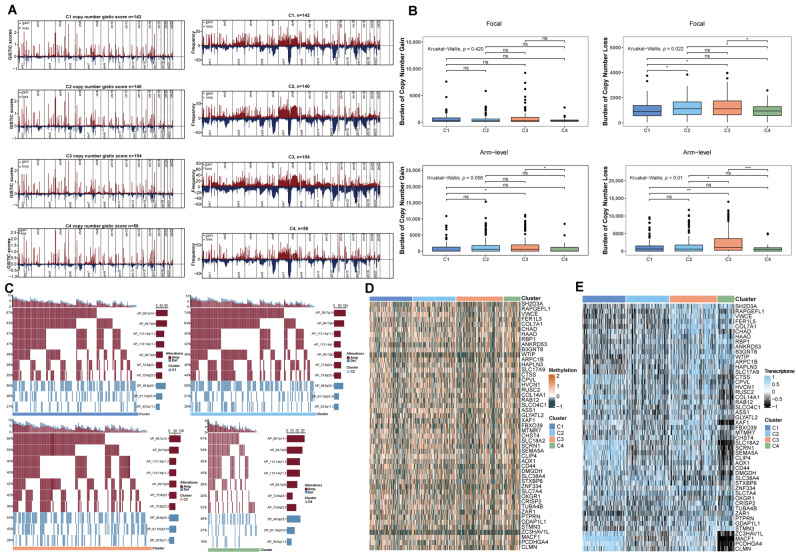
Copy number variations and epigenetic alterations across distinct PRAD clusters. (**A**) Copy number profiles across the four PRAD clusters within the TCGA-PRAD cohort (the left column indicates GISTIC scores, and the right column displays alteration frequencies). Red represents amplification, and blue represents deletion. Genomic loci are ordered according to their chromosomal positions, ranging from chromosomes 1 to 22. (**B**) Distribution of focal and arm-level copy number alterations within each PRAD cluster. Statistical differences among the groups were evaluated using the Kruskal–Wallis test. *: *p* < 0.05, **: *p* < 0.01, ***: *p* < 0.001; ns: not significant (*p* > 0.05). (**C**) Amplification and deletion plots detailing significant CNV regions across the four PRAD clusters in the TCGA-PRAD cohort, with chromosomal loci sorted by decreasing frequencies of alterations (amplifications or deletions). (**D**,**E**) Heatmaps showing the expression of genes significantly associated and all differentially expressed in the DNA methylation (**D**) and transcriptome (**E**) expression profiles of the four regulator-defined PRAD clusters in the cohort.

Given that somatic mutations and epigenetic dysregulation are integral to cancer pathogenesis, we investigated the CNV and DNA methylation profiles across the four regulator-defined PCa subtypes. Initially, GISTIC scores and CNV frequencies were determined for each subtype (Figure 4A). Tumors across all subtypes exhibited a predisposition toward copy number amplifications, notably on chromosomes 7–9; however, the C3 cohort demonstrated a significantly higher frequency of deletions relative to the other subtypes. As illustrated in Figure 4B, the C1 subtype displayed the lowest burden of both focal (<50% of a chromosome arm) and broad (≥50% of a chromosome arm) CNVs compared to the other groups (focal amplification: *p* = 0.420; focal deletion: *p* = 0.022; broad amplification: *p* = 0.056; broad deletion: *p* < 0.01). Conversely, C3 tumors harbored a markedly higher burden of both copy number gains and losses. These elevated structural variations denote pronounced genomic instability within the C3 subtype, aligning with its associated poor clinical outcomes. Furthermore, waterfall plot analysis delineated 10 specific chromosomal loci exhibiting significant differential amplification and deletion patterns among the subtypes (Figure 4C).

Beyond the canonical role of DNA CpG methylation in regulating gene expression during development and cellular homeostasis, its aberrant patterns serve as a critical epigenetic driver in oncogenesis. Accordingly, we evaluated the interplay between DNA methylation profiles and transcriptomic profiles within the training cohort. By integrating differential methylation analysis with RNA-seq data from the TCGA-PRAD cohort, we assessed epigenetic regulatory networks across the subtypes. We identified 49 genes exhibiting a significant negative correlation between methylation status and gene expression, wherein promoter hypomethylation corresponded with upregulated transcript levels (Figure 4D,E). Given that DNA methylation loss can facilitate immune evasion in tumors characterized by high mutational and copy number burden [34], these epigenetic alterations within the TCGA-PRAD dataset provide critical mechanistic insights into the divergent prognoses observed among our defined subtypes. Having demonstrated that these four histone modification regulator-defined subtypes possess distinct genomic, epigenetic, and prognostic landscapes, we subsequently sought to translate these multidimensional findings into a robust, clinically applicable prognostic model.

### 3.5. Construction of a Histone Modification Regulator-Guided HIS_score and Model Validation

**Figure 5:**
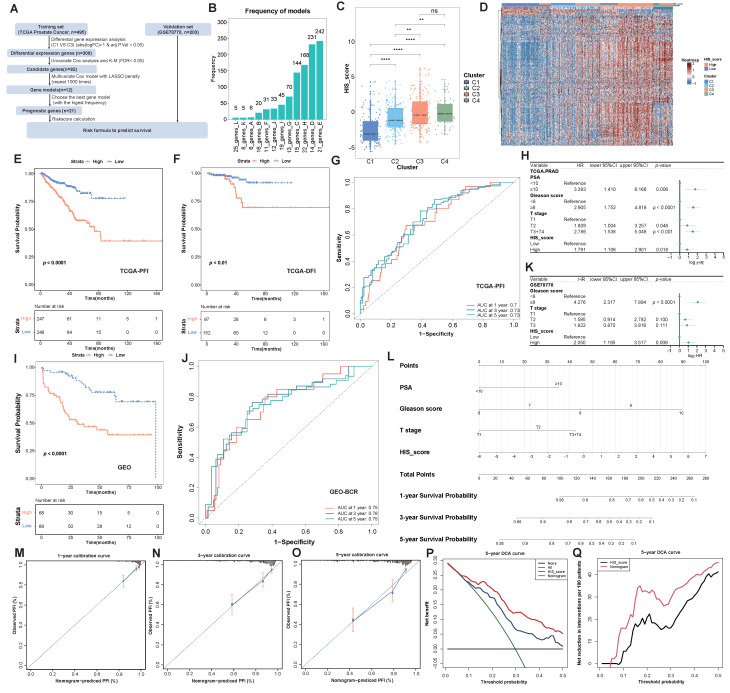
Construction and clinical relevance of the histone modification regulator-guided HIS_score model. (**A**) Flow diagram illustrating the development of the histone modification regulator-guided prognostic model. (**B**) Generation of 12 candidate gene signatures through 1000 repetitions. (**C**) HIS_score distribution of patients in the four PRAD clusters. (**D**) Heatmap showing DEGs across the PRAD clusters in the TCGA-PRAD cohort. The HIS_score and the four PRAD clusters are annotated for each sample. (**E**,**F**) Kaplan–Meier survival curves for PFI (**E**) and DFI (**F**) in the TCGA-PRAD cohort, stratified by the HIS_score. (**G**) Predictive value of the HIS_score for the 1-, 3-, and 5-year PFI rates of PCa patients in the TCGA-PRAD cohort. (**H**) Forest plot summarizing the multivariable Cox proportional hazards regression analysis for the HIS_score and clinicopathological characteristics (PSA, Gleason score, and T stage) within the TCGA-PRAD cohort. (**I**) Kaplan–Meier survival curves for DFS in the GSE70770 validation cohort, stratified by the HIS_score. (**J**) Predictive value of the HIS_score for the 1-, 3-, and 5-year DFS rates of PCa patients in the GSE70770 cohort. (**K**) Forest plot depicting univariable and multivariable Cox regression analyses of the HIS_score and clinical parameters (age, PSA, Gleason score, and T stage) in the GSE70770 cohort. (**L**) Nomogram integrating the HIS_score with standard clinical characteristics to predict 1-, 3-, and 5-year PFI probabilities for patients in the TCGA-PRAD cohort. (**M**–**O**) Calibration curves of the nomogram for predicting 1- (**M**), 3- (**N**), and 5-year (**O**) PFI in the TCGA-PRAD cohort. (**P**,**Q**) DCA of the nomogram based on the HIS_score for predicting 5-year outcomes. **: *p* < 0.01, ****: *p* < 0.0001; ns: not significant (*p* > 0.05).

Although our initial analysis successfully defined four distinct histone modification regulator expression subtypes (C1–C4) with significantly divergent prognoses—wherein C1 and C3 exhibited the most and least favorable outcomes, respectively (Figure 2D,E)—translating these findings into clinical practice necessitates a quantitative tool for individual risk stratification. To address this, we aimed to construct a robust risk-scoring model that captures the downstream transcriptomic consequences of these histone modification regulator-defined subtypes. Given that C1 and C3 represent the two extremes of our prognostic spectrum, we performed differential gene expression analysis between these two subtypes (Figure 5A, Supplementary Table S5). To isolate the optimal prognostic features, the resulting DEGs were subjected to univariate Cox regression and Kaplan–Meier survival analyses, followed by least absolute shrinkage and selection operator (LASSO) penalization analysis. Following 1000 rigorous iterations of LASSO regression with 10-fold cross-validation, 12 distinct gene combinations (models) were generated. Among these, a 21-gene signature emerged with the highest frequency (242/1000) and was consequently selected as the final prognostic signature (Supplementary Table S6, Figure 5B). Utilizing these 21 core genes, we established a novel histone modification-related predictive model, designated as the HIS_score. To enhance biological interpretability, we annotated the selected genes based on their established or emerging roles in PCa biology and histone modification-related pathways (detailed in the Discussion section). Briefly, at least six genes (including SULT1E1, COL11A2, MXD3, PIF1, TTLL10, and SLC9A3) demonstrated direct or indirect links to chromatin regulation, extracellular matrix remodeling, metabolic reprogramming, or androgen signaling. Several other components have been independently validated as PCa prognostic biomarkers in recent studies, aligning with the GSVA-enriched pathways (AR, cell cycle, and metabolism) observed across the subtypes. This functional annotation supports the prioritization of these specific genes over other correlated DEGs via our data-driven LASSO approach.

Box plot analysis revealed that C3 patients exhibited a significantly higher HIS_score compared to the other subtypes, whereas C1 patients displayed a relatively lower HIS_score (Figure 5C). Further stratification by common clinical characteristics revealed distinct HIS_score distributions across various patient subgroups (Supplementary Figure S2A–F). Subsequently, the patients were dichotomized into high- and low-score groups based on the median HIS_score cut-off value. A heatmap illustrating the expression profiles of these DEGs across the different subtypes (Figure 5D) demonstrated that the model could accurately discriminate patients within the TCGA-PRAD cohort. Kaplan–Meier analyses indicated that compared to the low-score group, the high-score group experienced significantly shorter PFI (*p* < 0.0001, Figure 5E) and DFI (*p* < 0.01, Figure 5F). Furthermore, the areas under the ROC curves for PFI at 1, 3, and 5 years were all greater than or equal to 0.70 (Figure 5G), confirming the reliability of the HIS_score as a predictive model. Univariate and multivariate Cox regression analyses further established that a high HIS_score served as an independent prognostic factor for PCa patients in the TCGA-PRAD dataset (univariate: *p* < 0.0001, HR = 2.996, 95% CI = 1.904–4.715; multivariate: *p* = 0.035, HR = 1.690, 95% CI = 1.038–2.750; Figure 5H). Crucially, its continued significance after adjusting for standard clinicopathological parameters—including PSA, Gleason score, and T stage—statistically demonstrates that the HIS_score contributes unique and supplementary prognostic variance beyond what conventional metrics provide. To independently validate the prognostic utility of this 21-gene signature, we calculated the HIS_score for each sample in the external GSE70770 validation cohort. Consistent with our training cohort findings, survival analysis demonstrated that patients in the high-score group had a significantly shorter DFS time than those in the low-score group (*p* < 0.001, Figure 5I). The corresponding ROC analysis yielded AUC values for DFS at 1, 3, and 5 years of 0.75, 0.76, and 0.75, respectively (Figure 5J), reaffirming the model’s capability to predict PCa recurrence or progression. Cox regression analyses within the GSE70770 cohort confirmed the significant association between the HIS_score and DFS (univariate: *p* < 0.001, HR = 2.484, 95% CI = 1.486–4.154; multivariate: *p* = 0.019, HR = 1.964, 95% CI = 1.119–3.446; Figure 5K).

We recognize that conducting unsupervised clustering and DEG identification on the entire TCGA-PRAD cohort prior to LASSO modeling may raise theoretical concerns regarding data leakage. However, this clustering step was intentionally exploratory and biology-driven, designed to prioritize transcriptomic alterations most germane to histone modification regulator dysregulation. Crucially, all penalized regression and model selection steps incorporated repeated 10-fold cross-validation, and the robustness of the final model is strongly corroborated by its consistent and independent performance in the external GSE70770 cohort.

To optimize predictive accuracy and clinical utility, we integrated the HIS_score with established clinicopathological factors (including T stage, PSA, and Gleason score) using the TCGA-PRAD dataset to formulate a prognostic nomogram for estimating PFI probabilities at various time points. As depicted in Figure 5L, higher cumulative scores derived from the nomogram corresponded to progressively lower 1-year, 3-year, and 5-year PFI rates. Calibration curves verified the reliability of the nomogram, demonstrating high concordance between the nomogram-predicted survival probabilities and the actual clinical observations (Figure 5M–O). Furthermore, DCA was employed to evaluate the net clinical benefit of the nomogram relative to individual predictive models (Figure 5P,Q). Unlike traditional performance metrics that solely assess statistical discrimination, DCA visually confirmed that integrating the HIS_score with standard clinical variables translates into a superior net clinical benefit across a wide spectrum of threshold probabilities. These findings, coupled with the multivariate independence of the HIS_score, substantiate that this novel metric introduces a meaningful, statistically tested layer of risk stratification, reinforcing the predictive efficacy and translational potential of the derived nomogram.

**Figure 6:**
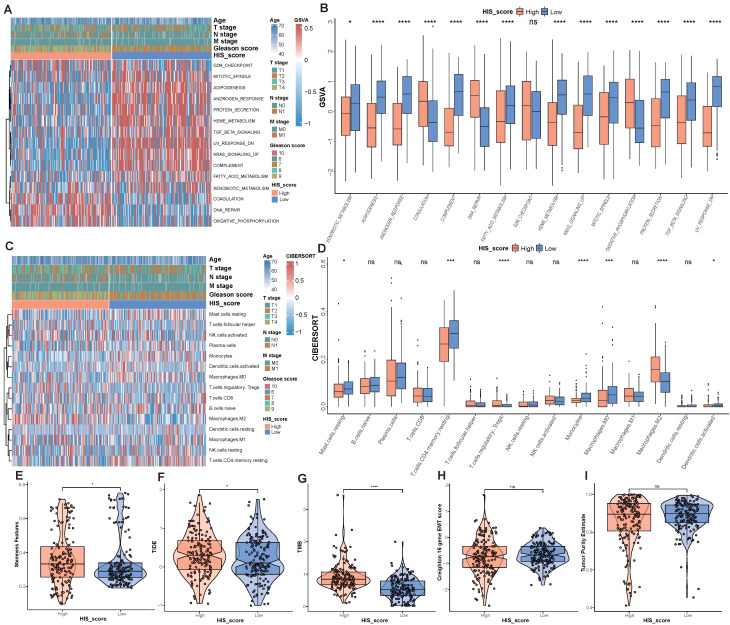
Biological and immune features in PCa patients stratified by the HIS_score. (**A**) Heatmap illustrating the GSVA enrichment scores of hallmark gene sets for patients in the high and low HIS_scores subgroups within the TCGA-PRAD cohort. Red indicates pathway activation, while blue denotes pathway inhibition. The HIS_score and clinical characteristics (age, TNM stage, and Gleason score) of the TCGA-PRAD cohort are provided as sample annotations. (**B**) Box plots comparing the GSVA scores between the high and low HIS_score subgroups. The boxes represent the interquartile range (25th to 75th percentiles), the horizontal lines indicate the median, the whiskers extend to 1.5 times the interquartile range, and the black dots represent outliers. (**C**) Results of cell type enrichment analysis in the high and low HIS_score subgroups, as estimated by the CIBERSORT algorithm. The HIS_score and clinical characteristics (age, TNM stage, and Gleason score) of the TCGA-PRAD cohort are provided as sample annotations. (**D**) Box plots comparing CIBERSORT-derived immune cell fractions between the high and low HIS_score subgroups. The boxes represent the interquartile range (25th to 75th percentiles), the horizontal lines indicate the median, the whiskers extend to 1.5 times the interquartile range, and the black dots represent outliers. (**E**–**I**) Distributions of stemness scores (**E**), TIDE scores (**F**), TMB scores (**G**), EMT scores (**H**), and tumor purity (**I**) in the high and low HIS_score subgroups of the TCGA-PRAD cohort. Statistical differences between the two groups were evaluated using the Wilcoxon rank-sum test. *: *p* < 0.05, ***: *p* < 0.001, ****: *p* < 0.0001; ns: not significant (*p* > 0.05).

Finally, we evaluated the therapeutic sensitivities, enriched biological pathways, and tumor immune infiltration landscapes of the high- and low-score groups. Mirroring the phenotype of C3 patients, the high-score group exhibited lower IC50 values for docetaxel, doxorubicin, and methotrexate (Supplementary Figure S3). Drug sensitivity estimates derived from the GDSC database using the pRRophetic algorithm indicated differential IC50 profiles across both the regulator-defined subtypes and the HIS_score risk groups. Since these represent in silico predictions based on cell line models, they should be interpreted cautiously; tumor heterogeneity, microenvironmental dynamics, and the absence of direct drug response data in this specific cohort may limit their direct clinical recapitulation. They serve primarily as hypothesis-generating insights to guide future preclinical or clinical validation. Pathway analysis revealed that the high HIS_score group was predominantly enriched in processes such as oxidative phosphorylation, coagulation cascades, and DNA repair mechanisms (Figure 6A,B). Furthermore, the infiltration scores for immunosuppressive Treg cells and M2 macrophages were significantly elevated in the high HIS_score group relative to the low-score group (Figure 6C,D). Correspondingly, this high-risk group exhibited elevated stemness features, TMB, and TIDE scores (Figure 6E–I), indicating higher malignancy and immunogenicity, as well as greater sensitivity to immune therapy. Based on these analyses, our HIS_score model, derived from histone modification regulator-defined subtypes, can distinguish PCa subgroups with different biological and immune characteristics.

## 4. Discussion

PCa exhibits a high incidence rate, and a subset of patients receiving localized therapies experience poor clinical outcomes and high mortality rates, highlighting the need for early intervention. Currently, effective and reliable clinical risk stratification biomarkers for PCa remain under investigation. Studies have shown that global changes in histone modifications are associated with patient prognosis, yet no related prognostic tool has been translated into routine clinical practice [13,35]. The clinical management of PCa is increasingly contingent upon precise molecular profiling. Beyond traditional clinicopathological parameters, the identification of treatment-related biomarkers and critical driver genes remains paramount for optimizing personalized therapeutic strategies. Recent investigations have significantly expanded this landscape, elucidating the roles of specific driver mutations and novel prognostic panels in determining treatment responsiveness and clinical trajectories in PCa [36,37,38]. Contextualized within these advancements, our proposed HIS_score—functioning essentially as a regulator-guided downstream signature—aligns cohesively with the current shift towards biomarker-driven oncology. Through precise risk stratification based on PFI outcomes, the HIS_score synergizes with currently recognized PCa driver genes. Ultimately, this delivers a multidimensional assessment tool that optimizes individualized clinical decision-making and ongoing disease surveillance.

In our study, we first performed unsupervised consensus clustering based on the transcriptional expression levels of 67 carefully curated histone modification regulators. This approach enabled us to identify four distinct expression patterns (PRAD clusters C1–C4) associated with significant differences in patient prognosis, biological functions, mutational characteristics, and therapeutic sensitivities. Both PFI and DFI outcomes were significantly more favorable in C1 patients compared to the other subtypes. Moreover, we observed that the histone modification genes SMYD5, SUV39H1, SIRT3, SIRT6, SIRT7, HDAC10, KAT2A, SIRT4, and HDAC8 were significantly upregulated in the C2–C4 patients with poorer prognoses compared to C1 patients, where their expression remained low. This suggests that these histone modification genes may affect the prognosis of PCa. Among them, SUV39H1 [39], SIRT6 [40], and SIRT7 [41] have been shown to mediate the proliferation and migration of PCa cells, and their overexpression can promote disease progression. Notably, the downregulation of SIRT7 expression can also increase the sensitivity of PCa cells to docetaxel and radiation therapy [41], providing a potential therapeutic target for drug-resistant PCa. This finding is consistent with the more favorable prognosis observed in C1 patients. Importantly, screening for small-molecule therapeutics using the GDSC database revealed that classifying patients by histone modification regulator expression patterns could significantly stratify their sensitivity to various chemotherapy drugs, further supporting our findings and providing insights for the development of new treatment strategies.

We further characterized the differences in biological functions and tumor immunity among the subtypes. Regarding biological characteristics, both C1 and C3 patients exhibited suppressed metabolic pathways alongside upregulated cell proliferation/cycle-related pathways, while C2 and C4 patients displayed an inverse pattern. This suggests that targeting the cell cycle may be a potential therapeutic strategy for C1 and C3 patients, whereas metabolic interventions may be effective for C2 and C4 patients. Specifically, we found that the AR pathway was highly activated in C1 and C2 patients, but the scores in C3 patients were significantly lower than in other subtypes. This profound divergence in AR signaling offers a critical mechanistic explanation for the cell cycle paradox observed between the C1 and C3 subtypes. The C3 subtype, characterized by a blunted AR coupled with high cell cycle activity, strongly mirrors the biological hallmarks of lineage plasticity and neuroendocrine differentiation (NED). Emerging evidence demonstrates that prostate tumors can evade androgen suppression by transdifferentiating into highly aggressive, AR-null neuroendocrine prostate cancer (NEPC) [42]. This lineage reprogramming is intimately governed by epigenetic dysregulation, particularly involving histone modifiers [43]. Therefore, C3 tumors likely represent a transition toward this neuroendocrine-like, androgen-independent phenotype. By bypassing AR dependency, they render standard androgen receptor signaling inhibitors ineffective, which comprehensively explains the dismal clinical outcomes of C3 patients despite their highly proliferative state [44]. Conversely, the C1 subtype maintains a robust AR signaling axis alongside an active cell cycle. This indicates that C1 tumors largely preserve their luminal epithelial identity and intrinsic AR dependency. Consequently, despite being highly proliferative, C1 tumors remain susceptible to canonical androgen-targeted therapies, elucidating their comparatively superior prognosis. Notably, complement pathway activation was significantly higher in C1 patients than in the other subtypes. The complement system promotes the phagocytic activity of macrophages [45,46]. Additionally, immune analysis revealed a high abundance of monocytes in C1 patients, which tended to differentiate into the M1 phenotype. M1 macrophages are characterized by their immunotoxic and phagocytic capabilities [47]. Therefore, we hypothesize that the synergistic effect between the highly activated complement pathway and the infiltration of M1 macrophages may be the primary driver of the favorable clinical outcomes observed in C1 patients. Furthermore, we found that although CD8+ T cell infiltration was abundant in C4 patients, immunosuppressive Treg cells were also significantly enriched, whereas they were sparse in other subtypes. Meanwhile, macrophages in C4 patients favored M2 polarization. Combined with the high TIDE score in C4 patients, we speculate that the cytotoxic lymphocytes in this subgroup are in an exhausted state, allowing the tumor to evade immune surveillance and leading to a poor response to immunotherapy.

Genomic profiling revealed an elevated burden of copy number gains and losses in the C3 subgroup. Specific CNVs shape tumor phenotypes and disease progression, thereby correlating strongly with patient prognosis. In various malignancies, an increased copy number burden is indicative of poorer clinical outcomes [48,49]. Additionally, previous studies have confirmed that an augmented copy number burden in PCa is associated with increased disease-specific mortality [47]. Therefore, the relative absence of such extensive genomic alterations in C1 tumors further substantiates why these patients have the most favorable prognosis.

Considering the significant differences across PCa subtypes defined by histone modification regulator expression patterns, we established a quantitative scoring model, the HIS_score. This 21-gene signature, derived from the transcriptomic differences between the extreme subtypes (C1 versus C3), aims to capture the downstream transcriptomic consequences of histone modification regulator dysregulation for clinical application. Compared to their low-scoring counterparts, high-scoring patients had a significantly worse prognosis; notably, C1 patients generally exhibited lower scores, confirming our earlier observation of their superior survival outcomes. Importantly, univariate and multivariate Cox regression analyses confirmed that the HIS_score serves as an independent prognostic factor for PCa. Incorporating the score into a clinical nomogram yielded robust predictive performance for 1-year, 3-year, and 5-year PFI. More importantly, as substantiated by the multivariate models and DCA, the HIS_score does not merely parallel standard variables like Gleason score or PSA; rather, it introduces a supplementary layer of risk stratification. This confirms that the downstream transcriptomic consequences of epigenetic dysregulation carry independent clinical weight, making our nomogram a highly valuable tool for facilitating personalized clinical treatment decisions.

We acknowledge that fitting a 21-gene signature in a cohort of 495 patients introduces analytical complexity, and an AUC of 0.75 in the external GSE70770 cohort requires contextualization. Predicting post-RP recurrence is historically challenging due to disease heterogeneity. Within the current landscape of PCa prognostic models, an external validation AUC of 0.75 is highly competitive. For reference, the 7-gene immune-related signature reported by Lv et al. [7] and the apoptosis-associated model developed by Zhang et al. [8] yield comparable validation AUCs, typically ranging from 0.70 to 0.76. Furthermore, the widely adopted 22-gene Decipher genomic classifier generally reports AUCs between 0.75 and 0.79 for predicting similar postoperative endpoints in independent cohorts [50]. Expanding this comparison to other established commercial tools, validation studies for the Prolaris Cell Cycle Progression (CCP) score and the Oncotype DX Genomic Prostate Score (GPS) demonstrate comparable prognostic discrimination, with post-treatment performance metrics (C-indices or AUCs) consistently clustering around 0.70 to 0.78 [51,52]. Beyond statistical parity, these signatures evaluate different biological axes. While assays like Prolaris focus heavily on cellular proliferation, the HIS_score functions as a regulator-guided downstream signature. By capturing the transcriptional sequelae of epigenetic dysregulation, it provides a distinct biological lens that could serve as a complementary layer of risk stratification. Viewed against these established academic and commercial benchmarks, the predictive performance of the HIS_score aligns well with state-of-the-art tools, supporting its practical clinical viability despite the model’s multi-gene complexity.

Although the use of an independent external validation cohort (GSE70770) strengthens the reliability of our HIS_score model, several important limitations warrant consideration. First, notable inter-cohort heterogeneity exists between the TCGA-PRAD and GSE70770 datasets. These datasets were generated using distinct technical platforms (RNA-seq versus microarray), represent diverse patient populations, and differ in the availability of key clinical variables (e.g., N and M stage information is largely unavailable in GSE70770). Furthermore, the outcome definitions vary slightly (PFI/DFI in TCGA-PRAD versus DFS in GSE70770), reflecting the well-recognized endpoint heterogeneity inherent in post-RP PCa studies. Consistent with the established clinical trajectory of post-RP patients, the prognostic events captured within our analysis were predominantly driven by BCR. Although isolated BCR does not universally translate to cancer-specific mortality, its accurate prediction through tools like the HIS_score remains clinically essential for identifying candidates who may benefit from early salvage interventions. Nevertheless, these endpoints are conceptually similar, and our model demonstrated consistent prognostic performance across both cohorts. Second, although the model development process was data-driven and incorporated rigorous measures to minimize overfitting—such as deriving candidate genes exclusively from the two subtypes with the most divergent prognoses (C1 and C3) and performing 1000 iterations of LASSO regression with 10-fold cross-validation solely in the training set—we acknowledge that the current validation strategy exhibits methodological constraints. Due to the scarcity of high-quality, publicly available transcriptomic datasets featuring PCa patients who underwent RP and complete long-term survival data (PFI/DFS), our external validation was restricted to a single independent cohort (GSE70770). Furthermore, additional internal validation techniques, such as bootstrap resampling or C-index comparisons across different baseline models, were not performed. Given the complexity of a 21-gene signature, broader validation across multiple independent, multicenter cohorts is essential to fully establish its robustness, stability, and reproducibility before making definitive clinical claims. Third, this study relied on publicly available anonymized datasets. While predefined inclusion/exclusion criteria minimized selection bias, certain missing clinical variables were addressed using complete-case analysis in Cox models. To enhance reproducibility, detailed analytical parameters are provided in the Methods section and Supplementary Materials; code deposition on public repositories such as GitHub is planned for future work. Fourth, we acknowledge a fundamental biological limitation regarding our reliance on transcriptomic data. Our study evaluates the mRNA expression levels of histone-modifying enzymes rather than directly assessing functional histone marks (e.g., H3K27ac, H3K27me3) via ChIP-seq or comparable epigenomic profiling techniques. Since mRNA abundance does not always linearly correlate with protein translation or dynamic enzymatic activity, our approach serves as an indirect surrogate. Rather than defining precise epigenomic landscapes, our analysis aims to capture the downstream transcriptomic consequences associated with the dysregulation of the histone modification machinery. While bulk RNA-sequencing data offer higher accessibility and facilitate the translation of such gene signatures into feasible clinical prognostic tools, future multi-omics studies integrating direct epigenomic profiling are necessary to fully substantiate these mechanistic links. Finally, as an exploratory bioinformatics study, our goal was to translate global transcriptional patterns of histone modification regulators into a clinically applicable tool. The HIS_score was deliberately constructed from DEGs between histone modification regulator-defined subtypes rather than being limited to the regulators themselves. This strategy captures the broader downstream transcriptomic consequences of histone modification regulator dysregulation while remaining firmly grounded in epigenetic biology.

Although several HIS_score genes (e.g., SULT1E1, MXD3, PIF1) have established links to chromatin regulation and PCa biology, systematic functional validation through gain- and loss-of-function experiments in cell lines and in vivo models is required. Such limitations are common in epigenetic prognostic modeling and underscore the need for integrative multi-omics and experimental studies. These limitations do not diminish the promising clinical utility of the HIS_score and its associated nomogram but instead highlight important directions for future refinement.

To systematically ascertain whether the 21 genes comprising the HIS_score signature act as direct mediators of histone modification or downstream effectors, we initially evaluated those possessing established mechanistic connections to the chromatin regulatory machinery. Notably, at least six of these genes are directly involved in histone modifications or closely linked chromatin pathways. For instance, SULT1E1 expression is upregulated by HDAC inhibitors in breast epithelial cell models, indicating that its transcription is repressed by histone deacetylation [53]; it might concurrently serve as a transcriptional mediator during PCa progression [54]. Similarly, COL11A2 is upregulated following HDAC inhibitor treatment during differentiation, implicating an epigenetic regulatory axis in extracellular matrix remodeling [55]. MXD3, as a downstream effector, heterodimerizes with MAX and recruits the Sin3-HDAC corepressor complex to promote local histone deacetylation and transcriptional silencing [56]. PIF1 functions as a bidirectional regulatory node: its transcription is governed by chromatin accessibility dictated by histone demethylases, whereas its enzymatic activity undergoes post-translational fine-tuning via NuA4-mediated acetylation, contributing to genomic stability amid altered epigenetic landscapes [57,58]. Furthermore, TTLL10 directly modifies nucleosome assembly protein 1 (NAP1), providing an enzymatic connection to chromatin architecture [59]. Additionally, SLC9A3 has been validated as a methylation-driven prognostic biomarker in PCa, where its hypermethylation is distinctly linked to disease recurrence [60]. Collectively, these findings offer compelling evidence that a substantial subset of the HIS_score signature is actively engaged in histone modification or chromatin-associated pathways, rather than representing mere passive byproducts of tumor biology.

Beyond this mechanistic core, several other signature genes have proven to be robust prognostic biomarkers for PCa across independent cohorts, although their direct connections to histone modifications require further characterization. Specifically, AMH was characterized as a novel biomarker for aggressive PCa in a prospective RP cohort, demonstrating that diminished serum AMH levels correlate with disease aggressiveness, whereas elevated levels predict improved DFS [61]. HAGHL, which encodes a glyoxalase II-like enzyme, has been integrated into metabolism-based prognostic models, exhibiting increased expression concomitant with PCa malignant progression [62]. KRTAP5-1 emerged as a highly influential prognostic factor in machine learning-driven risk classification models for PCa [63]. The transporters SLC6A14 (amino acid) and SLC39A5 (zinc) exhibit dysregulation during prostate tumorigenesis, aligning with the critical roles of zinc homeostasis and amino acid transport in PCa metabolic progression [64,65]. Finally, ALB serves as an established systemic prognostic factor in metastatic castration-resistant PCa, where hypoalbuminemia independently predicts adverse survival outcomes [66,67]. Consequently, while direct mechanistic evidence connecting these genes to histone modifications continues to accumulate, their robust prognostic performance and alignment with key enriched pathways (AR, cell cycle, and metabolism) underscore their biological relevance. This substantiates their selection via LASSO regression based on the differential expression between the C1 and C3 subtypes.

While our regulator-guided framework effectively captures the broader transcriptional consequences of epigenetic dysregulation, the biological coherence of the final 21-gene signature is not uniformly tethered to canonical histone pathways. It is important to recognize that our feature selection heavily relied on LASSO regression. By its design, this penalization method minimizes collinearity to maximize independent predictive value, inherently favoring an optimal mathematical combination of genes across diverse, non-overlapping biological processes rather than clustering within a single functional cascade. This algorithmic prioritization largely explains why certain structural components or metabolic indicators—such as ALB, GPT, or the KRTAP family—were selected despite lacking classical epigenetic roles; they likely represent downstream “passenger” alterations or systemic metabolic sequelae stemming from the profound epigenetic reprogramming observed in the C3 phenotype. For a minor subset of the signature—comprising CCDC28B, GPT, DNASE1L2, KRTAP5-9, KRTAP5-10, MSLNL, NKAIN4, CCDC114, and RPE65—direct evidence linking them to histone modifications within the context of PCa remains sparse. Nonetheless, their repeated identification in bioinformatics-derived prognostic models implies underlying biological significance, warranting deeper functional investigation. Clarifying whether these genes represent downstream targets of epigenetic reprogramming or operate as independent prognostic modules remains a critical avenue for future research. Conclusively resolving these regulatory dynamics and confirming direct epigenetic modulation will ultimately require multi-omics investigations incorporating matched chromatin accessibility data, such as ATAC-seq or ChIP-seq.

Overall, by coupling the unsupervised clustering of histone modification regulators with the subsequent construction of a downstream gene signature, we successfully developed the 21-gene HIS_score model. Our analyses demonstrate that this model effectively predicts the post-RP prognosis of patients with PCa. Such stratification can assist clinicians in the early identification of high-risk patients, thereby facilitating the formulation and adjustment of personalized therapeutic strategies. However, certain limitations must be acknowledged. First, given the retrospective nature of this investigation utilizing public databases, future prospective cohort studies employing standardized inclusion criteria are required to validate the generalizability of these findings. Second, a proportion of the transcripts constituting the HIS_score lack functional characterization within the specific context of PCa. Although computational evidence and existing literature connect SULT1E1, COL11A2, MXD3, PIF1, TTLL10, and SLC9A3 to histone modification or allied chromatin regulatory networks, and genes like AMH, HAGHL, KRTAP5-1, SLC6A14, SLC39A5, and ALB possess independent prognostic value in PCa, the specific mechanistic contributions of members such as CCDC28B, GPT, DNASE1L2, KRTAP5-9, KRTAP5-10, MSLNL, NKAIN4, CCDC114, and RPE65 remain largely unresolved. Consequently, systematic gain- and loss-of-function experiments in PCa cell lines are necessary to validate the functional contribution of these genes to tumor progression. This experimental validation represents a critical prerequisite for translating the HIS_score signature into a clinically actionable diagnostic assay.

## 5. Conclusions

In summary, this study demonstrates that classifying PCa based on histone modification patterns has significant clinical relevance. The regulator-guided HIS_score, derived from differentially expressed genes between extreme subtypes, along with the novel nomogram, are promising exploratory tools that capture the downstream transcriptomic consequences of histone modification dysregulation. While broader validation is required before definitive clinical application, they provide a valuable proof-of-concept for predicting the prognosis of PCa patients after RP and may assist clinicians in developing personalized treatment plans in the future.

## Figures and Tables

**Table 1:** Histone modification genes are significantly associated with poor prognosis in PCa.

Gene	Modification	HR	*p* Value
EZH2	Methyltransferases	3.207	1.280 × 10^−5^
SETD4	Methyltransferases	2.005	6.533 × 10^−4^
SUV39H1	Methyltransferases	2.110	1.136 × 10^−3^
EHMT1	Methyltransferases	1.567	3.841 × 10^−2^
DOT1L	Methyltransferases	1.866	2.805 × 10^−3^
EHMT2	Methyltransferases	2.182	2.899 × 10^−3^
SETD5	Methyltransferases	1.793	6.439 × 10^−3^
SETD1B	Methyltransferases	1.658	1.581 × 10^−2^
N6AMT1	Methyltransferases	1.861	2.197 × 10^−2^
KDM2B	Demethylases	2.377	7.639 × 10^−3^
KDM6B	Demethylases	1.789	4.889 × 10^−2^
KAT2A	Acetyltransferases	2.757	4.900 × 10^−7^
HDAC7	Deacetylases	2.567	1.390 × 10^−5^
SIRT7	Deacetylases	2.065	3.771 × 10^−4^
HDAC10	Deacetylases	1.972	8.793 × 10^−4^
HDAC3	Deacetylases	2.079	3.116 × 10^−3^
HDAC6	Deacetylases	1.725	3.238 × 10^−2^
SIRT6	Deacetylases	1.743	1.055 × 10^−2^
HDAC11	Deacetylases	1.955	1.102 × 10^−2^
SIRT3	Deacetylases	1.901	1.113 × 10^−2^

## Data Availability

Data are available from the corresponding author if a justification for the requirement is provided.

## Supplementary Materials

The following supporting information can be downloaded at: https://www.mdpi.com/article/10.3390/biomedicines14061219/s1, Table S1: Baseline characteristics of the enrolled patients in each dataset; Table S2: List of the histone modification regulators; Table S3: Basic information of gene expression profiling series; Table S4: Effects of the expression levels of histone modification regulators on the survival of PCa patients; Table S5: Differentially expressed genes between patients in the C1 and C3 groups; Table S6: The 21-gene signature and formula for calculating the HIS_score; Figure S1: Differences in hazard ratios and drug sensitivities across the PRAD clusters; Figure S2: Associations between the HIS_score and clinical characteristics of PCa patients; Figure S3: Differences in drug sensitivity between the high and low HIS_score subgroups.

## Abbreviations

The following abbreviations are used in this manuscript:

PCa	Prostate cancer
RP	Radical prostatectomy
TCGA	The Cancer Genome Atlas
GEO	Gene Expression Omnibus
PSA	Prostate-specific antigen
PRAD	Prostate adenocarcinoma
UCSC	University of California, Santa Cruz
PFI	Progression-free interval
BCR	Biochemical recurrence
DFI	Disease-free interval
DFS	Disease-free survival
DEGs	Differentially expressed genes
CNV	Copy number variation
GDSC	Genomics of Drug Sensitivity in Cancer
ROC	Receiver operating characteristic
AUC	Area under the curve
GSVA	Gene set variation analysis
TIDE	Tumor immune dysfunction and exclusion
DCA	Decision curve analysis
EMT	Epithelial–mesenchymal transition
IC50	Half-maximal inhibitory concentration
TMB	Tumor mutational burden
AR	Androgen response
NED	Neuroendocrine differentiation
NEPC	Neuroendocrine prostate cancer
