# Supplementary material for: A Gene Feature Based on Histone Modifications Can Predict the Prognosis of Prostate Cancer
Source: Biomedicines. 2026 May 28;14(6):1219. doi: 10.3390/biomedicines14061219 (PMC13296647; doi:10.3390/biomedicines14061219)
Supplement: Supplementary file 1 [file biomedicines-14-01219-s001.zip › biomedicines-4179553-supplementary/Supplementary material.pdf]

Supplementary Materials

Supplementary Tables

Table S1. Baseline characteristics of the enrolled patients in each dataset.

Item	TCGA-PRAD cohort (n=495)	GSE70770 cohort (n=203)
**Age (mean ± SD)**	61.03±6.836	60.46±6.572
Unknown	0	92
**T stage (n, %)**		
T1	177 (35.8)	103(50.7)
T2	203(41.0)	72(35.5)
T3	109(22.0)	25(12.3)
T4	3(0.6)	0(0)
Unknown	3(0.6)	3(1.5)
**N stage (n, %)**		
N0	344(69.5)	
N1	78(15.8)	
Unknown	73(14.7)	
**M stage (n, %)**		
M0	453(91.5)	
M1	3(0.6)	
Unknown	39(7.9)	
**Gleason score (n, %)**		
1	0(0)	1(0.5)
5	0(0)	2(1.0)
6	45(9.1)	35(17.2)
7	246(49.7)	140(69.0)
8	63(12.7)	13(6.4)
9	137(27.7)	10(4.9)
10	4(0.8)	0(0)
Unknown	0(0)	2(1.0)

Note: This table summarizes the clinicopathological characteristics of patients included in the study. The inclusion criteria for both datasets comprised: (1) histologically confirmed primary PRAD; (2) RP as the primary surgical intervention; and (3) complete baseline clinical and survival records. Patients were excluded based on: (1) metastatic disease (M1) at initial diagnosis; or (2) missing essential clinicopathological metrics (PSA, Gleason score, or TNM stage) and survival outcomes. Both node-positive and node-negative individuals were eligible for inclusion. The final analysis included 495 patients from the TCGA-PRAD cohort and 203 patients from the GSE70770 cohort. In the GSE70770 cohort (n = 203), age data were missing or labeled as "Unknown" in 92 cases. The reported mean age was calculated based on only patients with available age information. For all prognostic analyses (unsupervised consensus clustering, differential expression, LASSO regression, Cox proportional hazards modeling, Kaplan–Meier survival analysis, and HIS_score construction/validation), complete-case analysis was performed using patients with sufficient gene expression and survival data who met the inclusion/exclusion criteria. No imputation was conducted.

The primary survival endpoint (DFS) and gene expression profiles were complete for all 203 patients in the final analysis.

Table S2. List of the histone modification regulators.

Acetyltransferases	Deacetylases	Methyltransferases	Demethylases
KAT5	HDAC9	SUV39H2	KDM6B
KAT2B	HDAC8	SUV39H1	KDM5B
KAT2A	HDAC7	SETD7	KDM5A
CREBBP	HDAC6	SETD6	KDM4D
EP300	HDAC5	SETD5	KDM3B
ELP3	HDAC4	SETD4	KDM2B
HAT1	HDAC3	SETD3	KDM1B
NCOA1	HDAC2	SETD2	PHF8
CLOCK	HDAC11	SETD1B	PHF2
GTF3C4	HDAC10	SETD1A	JMJD1C
	HDAC1	PRDM9	UTY
	SIRT7	SETDB2	
	SIRT6	SETDB1	
	SIRT5	EHMT2	
	SIRT4	EHMT1	
	SIRT3	NSD1	
	SIRT2	ASH1L	
	SIRT1	N6AMT1	
		SMYD5	
		SMYD4	
		SMYD3	
		SMYD2	
		SMYD1	
		DOT1L	
		EZH2	
		PRMT6	
		PRMT5	
		CARM1	

Table S3. Basic information of gene expression profiling series.

Datasets	Platform	Country	No. of patients	Survival
TCGA-PRAD	RNAseq	USA	495	PFI/DFI
GSE70770	GPL10558	UK	203	DFS

PFI: progression-free interval; DFI: disease-free interval; DFS: disease-free survival.

Table S4. Effects of the expression levels of histone modification regulators on the survival of PCa patients.

Gene name	Modification	HR	*P* value	logFC	adj.P.Val
EZH2	Methyltransferases	3.207	1E-05	1.504	1E-35
SETD4	Methyltransferases	2.005	7E-04	0.097	0.104
SUV39H1	Methyltransferases	2.110	0.001	0.085	0.096
EHMT1	Methyltransferases	1.567	0.038	0.243	4E-05
DOT1L	Methyltransferases	1.866	0.003	0.656	2E-16
EHMT2	Methyltransferases	2.182	0.003	0.282	8E-09
SETD5	Methyltransferases	1.793	0.006	0.039	0.622
SETD1B	Methyltransferases	1.658	0.016	0.125	0.067
N6AMT1	Methyltransferases	1.861	0.022	0.154	0.009
NSD1	Methyltransferases	0.251	0.011	-0.092	0.296
SMYD1	Methyltransferases	0.465	0.015	-1.067	0.011
ASH1L	Methyltransferases	0.317	0.004	-0.356	0.001
SETD7	Methyltransferases	0.454	9E-04	0.102	0.295
SETD3	Methyltransferases	0.565	0.025	-0.073	0.061
SMYD4	Methyltransferases	0.436	0.03	-0.441	5E-12
SETDB1	Methyltransferases	1.624	0.051	0.095	0.108
SETD2	Methyltransferases	1.572	0.061	-0.151	0.053
SUV39H2	Methyltransferases	0.712	0.102	0.363	2E-05
SETD1A	Methyltransferases	1.481	0.125	0.112	0.017
CARM1	Methyltransferases	1.795	0.131	-0.025	0.704
SMYD5	Methyltransferases	1.543	0.132	0.275	1E-10
SETDB2	Methyltransferases	1.382	0.137	-0.172	0.025
SMYD2	Methyltransferases	0.727	0.153	0.626	8E-11
SMYD3	Methyltransferases	1.312	0.191	0.683	1E-16
PRMT6	Methyltransferases	0.766	0.199	0.439	3E-06
PRMT5	Methyltransferases	1.309	0.352	0.159	0.03
SETD6	Methyltransferases	1.231	0.409	0.311	9E-07
PRDM9	Methyltransferases	1.103	0.723	0.21	0.166
KDM2B	Demethylases	2.377	0.008	-0.214	0.004
KDM5A	Demethylases	0.531	0.019	-0.27	8E-04
KDM4D	Demethylases	0.438	0.044	0.018	0.824
KDM6B	Demethylases	1.789	0.049	0.037	0.666
KDM1B	Demethylases	0.585	0.062	0.042	0.638
KDM3B	Demethylases	0.515	0.086	-0.199	0.011
PHF8	Demethylases	0.561	0.095	0.424	3E-05
PHF2	Demethylases	1.451	0.133	-0.115	0.203
UTY	Demethylases	0.655	0.169	-0.357	2E-04
KDM5B	Demethylases	1.440	0.172	0.306	6E-05
JMJD1C	Demethylases	0.639	0.251	-0.134	0.217
KAT2A	Acetyltransferases	2.757	5E-07	0.966	1E-25
ELP3	Acetyltransferases	0.306	0.003	-0.647	3E-22

CLOCK	Acetyltransferases	0.281	0.003	-0.22	0.006
KAT2B	Acetyltransferases	0.552	0.004	-0.396	6E-05
EP300	Acetyltransferases	0.436	0.03	-0.141	0.208
GTF3C4	Acetyltransferases	1.746	0.053	0.144	0.043
HAT1	Acetyltransferases	0.711	0.103	0.201	0.002
KAT5	Acetyltransferases	0.746	0.194	-0.185	2E-04
NCOA1	Acetyltransferases	1.447	0.201	-0.18	0.04
CREBBP	Acetyltransferases	1.341	0.21	-0.12	0.127
HDAC7	Deacetylases	2.567	1E-05	0.016	0.774
SIRT7	Deacetylases	2.065	4E-04	0.433	6E-11
HDAC10	Deacetylases	1.972	9E-04	0.891	6E-13
HDAC3	Deacetylases	2.079	0.003	0.235	2E-07
HDAC6	Deacetylases	1.725	0.032	0.139	0.013
SIRT6	Deacetylases	1.743	0.011	0.363	1E-07
HDAC11	Deacetylases	1.955	0.011	0.035	0.557
SIRT3	Deacetylases	1.901	0.011	0.102	0.032
HDAC9	Deacetylases	0.568	0.007	-0.795	2E-04
SIRT1	Deacetylases	0.544	0.019	-0.155	0.074
SIRT4	Deacetylases	0.574	0.026	0.185	0.037
HDAC1	Deacetylases	1.973	0.061	0.363	6E-07
SIRT2	Deacetylases	0.699	0.084	0.146	0.002
HDAC4	Deacetylases	1.568	0.108	-0.172	0.031
HDAC8	Deacetylases	1.509	0.132	0.318	1E-14
HDAC5	Deacetylases	1.363	0.141	-0.171	0.003
SIRT5	Deacetylases	1.49	0.167	-0.024	0.591
HDAC2	Deacetylases	0.805	0.441	0.144	0.055

Table S5. Differentially expressed genes between patients in the C1 and C3 groups.

Genes	log2FC	t	P Value	adj.P.Val	B
NOL12	1.002	20.96	3.31E-70	5.33E-66	149.06
NPEPL1	1.382	19.04	5.36E-61	2.87E-57	128.04
ALKBH6	1.188	18.93	1.77E-60	5.69E-57	126.85
UBXN11	1.113	17.95	8.14E-56	1.87E-52	116.21
SNRNP70	1.068	17.77	5.51E-55	9.02E-52	114.31
CCNL2	1.37	17.77	5.61E-55	9.02E-52	114.29
HDAC10	1.243	17.63	2.57E-54	3.44E-51	112.79
LUC7L	1.083	17.42	2.68E-53	3.07E-50	110.46
LIME1	1.665	17.25	1.60E-52	1.60E-49	108.69
EXD3	1.053	17.12	6.74E-52	6.37E-49	107.26
ATAD3B	1.249	17.08	1.04E-51	9.26E-49	106.84
SCNN1D	1.773	16.74	4.09E-50	3.13E-47	103.19
AGER	1.461	16.69	6.96E-50	5.09E-47	102.67
CHKB	1.219	16.43	1.02E-48	5.83E-46	100.01
LENG8	1.176	16.41	1.35E-48	7.23E-46	99.728

BGLAP	1.796	16.3	4.33E-48	2.17E-45	98.572
NEIL1	1.343	16.08	4.32E-47	1.87E-44	96.293
NOXA1	1.28	16.05	5.97E-47	2.46E-44	95.971
ZNF692	1.13	15.95	1.76E-46	6.58E-44	94.899
ARL6IP4	1.043	15.91	2.55E-46	9.11E-44	94.532
GPS2	1.138	15.86	4.40E-46	1.51E-43	93.99
ATG16L2	1.309	15.78	1.08E-45	3.48E-43	93.099
YJEFN3	2.047	15.65	4.04E-45	1.18E-42	91.793
MAMDC4	1.681	15.55	1.18E-44	3.32E-42	90.733
LCN12	1.53	15.39	6.21E-44	1.69E-41	89.085
EME2	1.515	15.37	7.83E-44	2.06E-41	88.855
MST1	1.56	15.35	9.98E-44	2.59E-41	88.615
KLHL17	1.035	15.32	1.28E-43	3.22E-41	88.367
TMEM86B	1.079	15.32	1.35E-43	3.33E-41	88.316
CSAD	1.298	15.28	1.97E-43	4.73E-41	87.94
DNAH17	1.077	15.17	6.27E-43	1.48E-40	86.793
DCST2	1.529	15.12	1.05E-42	2.42E-40	86.279
MAPK8IP3	1.205	15.09	1.46E-42	3.26E-40	85.955
CPT1B	1.622	15.05	2.09E-42	4.55E-40	85.599
MXD3	1.467	14.79	3.07E-41	5.91E-39	82.936
CCDC28B	1.061	14.74	5.29E-41	9.66E-39	82.398
C8G	1.267	14.7	8.03E-41	1.43E-38	81.984
CHTF18	1.101	14.66	1.25E-40	2.17E-38	81.543
TTLL3	1.277	14.58	2.65E-40	4.44E-38	80.8
KCNIP2	1.288	14.48	7.25E-40	1.17E-37	79.804
FGF17	2.31	14.45	1.07E-39	1.68E-37	79.415
AGAP6	1.146	14.39	1.95E-39	2.97E-37	78.823
PCP2	1.718	14.29	5.18E-39	7.57E-37	77.855
DRD4	1.558	14.27	6.53E-39	9.46E-37	77.625
COL11A2	1.673	14.26	7.39E-39	1.04E-36	77.503
EGFL8	1.457	14.24	8.84E-39	1.24E-36	77.326
CALML6	2.311	14.2	1.24E-38	1.68E-36	76.99
CDK3	1.473	14.1	3.63E-38	4.71E-36	75.926
HSF4	1.723	14.02	7.75E-38	9.81E-36	75.175
PRR22	1.196	13.84	4.92E-37	5.90E-35	73.344
SEC31B	1.551	13.81	6.60E-37	7.75E-35	73.052
SLC23A3	1.572	13.74	1.39E-36	1.57E-34	72.316
GSDMB	1.496	13.72	1.65E-36	1.84E-34	72.147
TMEM91	1.023	13.62	4.44E-36	4.81E-34	71.165
KIAA0895L	1.022	13.5	1.40E-35	1.44E-33	70.03
PRICKLE4	1.277	13.31	9.21E-35	8.70E-33	68.162
MEF2B	1.286	13.31	9.40E-35	8.82E-33	68.142
CRYGS	1.262	13.3	1.01E-34	9.37E-33	68.072
TNFRSF25	1.467	13.24	1.90E-34	1.68E-32	67.445

PABPC1L	1.717	13.24	1.97E-34	1.72E-32	67.41
GDPD3	1.338	13.15	4.42E-34	3.70E-32	66.608
PCSK4	1.095	13.1	7.29E-34	5.95E-32	66.113
PROCA1	1.121	13.07	1.04E-33	8.41E-32	65.76
INHA	1.587	13.03	1.48E-33	1.16E-31	65.415
GNRH1	1.37	13.02	1.58E-33	1.23E-31	65.347
PILRB	1.697	12.91	4.51E-33	3.33E-31	64.308
RNF207	1.282	12.88	6.06E-33	4.29E-31	64.016
LY6G5B	1.369	12.86	7.61E-33	5.32E-31	63.791
ITIH4	1.356	12.84	9.45E-33	6.52E-31	63.577
TMPRSS5	1.334	12.82	1.17E-32	8.04E-31	63.365
DCST1	1.343	12.8	1.31E-32	8.95E-31	63.25
LAT	1.557	12.79	1.47E-32	9.96E-31	63.136
LTB4R	1.25	12.75	2.24E-32	1.48E-30	62.72
AMY2B	1.49	12.65	5.88E-32	3.69E-30	61.767
HES4	1.394	12.63	6.76E-32	4.23E-30	61.629
PBX4	1.295	12.62	7.48E-32	4.64E-30	61.528
ANKRD23	1.164	12.62	7.65E-32	4.73E-30	61.506
SLC39A5	1.282	12.61	8.87E-32	5.46E-30	61.36
KIFC2	1.404	12.6	9.23E-32	5.66E-30	61.32
FAM166A	1.741	12.48	2.99E-31	1.76E-29	60.158
NTN5	1.521	12.48	3.02E-31	1.78E-29	60.146
ANKLE1	1.192	12.46	3.49E-31	2.01E-29	60.005
KCNAB3	1.348	12.46	3.70E-31	2.12E-29	59.946
CCDC17	1.377	12.32	1.38E-30	7.63E-29	58.641
ODF3B	1.255	12.3	1.65E-30	8.96E-29	58.466
TMPRSS9	1.468	12.23	3.27E-30	1.73E-28	57.788
MYO15B	1.407	12.22	3.47E-30	1.83E-28	57.731
LEAP2	1.243	12.13	8.30E-30	4.09E-28	56.867
CHRNA10	1.097	12.11	9.62E-30	4.70E-28	56.722
CYP2D6	1.241	11.97	3.52E-29	1.60E-27	55.437
GOLGA8A	1.841	11.92	5.78E-29	2.56E-27	54.948
HSD17B3	1.824	11.91	6.29E-29	2.77E-27	54.864
TSNAXIP1	1.044	11.85	1.15E-28	4.91E-27	54.27
GABBR1	1.077	11.8	1.80E-28	7.53E-27	53.824
HGFAC	2.102	11.79	2.04E-28	8.42E-27	53.701
SLC25A27	1.322	11.77	2.36E-28	9.70E-27	53.556
MC1R	1.188	11.76	2.56E-28	1.04E-26	53.476
MYCBPAP	1.129	11.68	5.48E-28	2.14E-26	52.722
UCN	1.207	11.55	1.81E-27	6.74E-26	51.54
PDE6C	1.283	11.53	2.21E-27	8.10E-26	51.345
RHPN1	1.112	11.5	2.72E-27	9.87E-26	51.138
LYG1	1.243	11.5	2.82E-27	1.02E-25	51.102
CCDC73	1.212	11.45	4.47E-27	1.59E-25	50.647

CRYBB3	1.365	11.44	4.91E-27	1.74E-25	50.555
KLHL11	-1.09	-11.4	5.80E-27	2.04E-25	50.389
MAPK15	1.803	11.4	6.76E-27	2.37E-25	50.238
GPR35	1.158	11.38	8.33E-27	2.90E-25	50.031
TRIM74	1.541	11.26	2.40E-26	7.90E-25	48.984
CCDC85B	1.014	11.24	3.10E-26	1.01E-24	48.73
TFAP2E	1.06	11.23	3.20E-26	1.04E-24	48.699
MSH5	1.082	11.17	5.41E-26	1.68E-24	48.182
TRIM73	1.255	11.16	6.01E-26	1.87E-24	48.077
ZDHHC11B	1.33	11.12	8.85E-26	2.70E-24	47.695
REC8	1.081	11.06	1.53E-25	4.54E-24	47.155
SLC25A34	1.151	11.04	1.71E-25	5.04E-24	47.042
CCDC78	1.817	11.04	1.77E-25	5.21E-24	47.007
REM2	1.29	11.04	1.85E-25	5.41E-24	46.968
SLC9A5	1.017	10.93	4.69E-25	1.30E-23	46.046
DOC2A	1.803	10.88	7.19E-25	1.95E-23	45.625
GPT	1.445	10.86	8.56E-25	2.31E-23	45.452
PLA2G4B	1.427	10.84	1.02E-24	2.73E-23	45.28
ALS2CL	1.253	10.79	1.60E-24	4.21E-23	44.834
DNLZ	1.155	10.77	2.01E-24	5.20E-23	44.609
CRYBG3	-1.02	-10.7	3.60E-24	9.14E-23	44.033
HPX	1.147	10.68	4.13E-24	1.04E-22	43.897
GOLGA8B	1.62	10.67	4.63E-24	1.17E-22	43.783
RAPH1	-1.06	-10.7	5.18E-24	1.29E-22	43.673
RASL10A	1.27	10.59	9.25E-24	2.27E-22	43.101
SLC16A8	1.423	10.58	9.89E-24	2.42E-22	43.035
CDRT4	1.036	10.54	1.51E-23	3.61E-22	42.615
DNASE1L2	1.303	10.5	2.10E-23	4.93E-22	42.29
UNC5CL	1.021	10.41	4.67E-23	1.05E-21	41.501
CRIP1	1.328	10.36	6.73E-23	1.50E-21	41.14
SPTBN5	1.109	10.34	8.10E-23	1.79E-21	40.958
ZNF296	1.087	10.31	1.02E-22	2.23E-21	40.727
ITGA2B	1.065	10.24	1.92E-22	4.11E-21	40.107
PAQR6	1.479	10.2	2.65E-22	5.58E-21	39.787
ACER2	-1.08	-10.2	2.67E-22	5.62E-21	39.779
STRC	1.491	10.17	3.42E-22	7.16E-21	39.535
SLC34A3	1.354	10.13	4.76E-22	9.81E-21	39.208
COL7A1	1.308	10.12	5.48E-22	1.12E-20	39.071
PROZ	1.592	10.03	1.15E-21	2.28E-20	38.341
IRF9	1.013	10.02	1.24E-21	2.46E-20	38.264
GRIN3B	1.781	10.01	1.40E-21	2.77E-20	38.143
FTCD	1.268	10	1.43E-21	2.81E-20	38.127
SLC7A9	1.216	10	1.43E-21	2.82E-20	38.123
PIF1	1.036	9.952	2.19E-21	4.22E-20	37.702

KRTAP5-9	1.266	9.885	3.84E-21	7.15E-20	37.15
CORO6	1.453	9.869	4.38E-21	8.14E-20	37.019
SRPK3	1.152	9.83	6.06E-21	1.11E-19	36.699
NPPA	1.414	9.783	8.94E-21	1.61E-19	36.315
RGS11	1.24	9.772	9.79E-21	1.75E-19	36.226
TNFRSF18	1.216	9.722	1.49E-20	2.58E-19	35.814
PLAC8L1	1.122	9.721	1.49E-20	2.59E-19	35.811
TTLL10	1.477	9.675	2.18E-20	3.73E-19	35.434
KRTAP5-1	1.654	9.656	2.54E-20	4.33E-19	35.283
CAPN12	1.085	9.64	2.91E-20	4.92E-19	35.151
IGFL4	1.318	9.58	4.76E-20	7.84E-19	34.665
GPC2	1.16	9.544	6.37E-20	1.03E-18	34.379
PIP5KL1	1.049	9.542	6.44E-20	1.04E-18	34.367
KCNT1	1.416	9.457	1.29E-19	2.02E-18	33.682
ERAS	1.305	9.444	1.43E-19	2.22E-18	33.583
MIOX	1.452	9.444	1.44E-19	2.24E-18	33.576
GNB3	1.156	9.349	3.08E-19	4.59E-18	32.825
KRTAP5-10	1.503	9.342	3.25E-19	4.84E-18	32.77
PDIA2	2.393	9.267	5.95E-19	8.62E-18	32.175
PNMA3	1.361	9.258	6.37E-19	9.17E-18	32.109
MLXIPL	1.15	9.175	1.24E-18	1.73E-17	31.454
SH2D6	1.226	9.115	1.99E-18	2.70E-17	30.985
BAIAP2L2	1.639	9.106	2.13E-18	2.88E-17	30.919
CAPN3	1.215	9.082	2.57E-18	3.45E-17	30.733
GIPR	1.419	9.065	2.93E-18	3.91E-17	30.605
TNNT2	1.39	9.045	3.44E-18	4.56E-17	30.446
HES7	1.3	9.032	3.81E-18	5.01E-17	30.347
FBXW12	1.173	8.999	4.92E-18	6.39E-17	30.094
IL4	1.322	8.984	5.53E-18	7.18E-17	29.979
C1QTNF4	1.153	8.979	5.77E-18	7.45E-17	29.937
LGALS4	1.339	8.923	8.95E-18	1.14E-16	29.505
AZU1	1.348	8.899	1.07E-17	1.36E-16	29.326
ASMT	1.182	8.842	1.68E-17	2.08E-16	28.886
TTC16	1.134	8.797	2.36E-17	2.90E-16	28.549
OCM	1.201	8.757	3.23E-17	3.91E-16	28.242
GALNT4	-1.03	-8.66	6.76E-17	7.90E-16	27.515
HAGHL	1.212	8.643	7.67E-17	8.93E-16	27.39
PRRT2	1.013	8.597	1.09E-16	1.25E-15	27.047
LTC4S	1.135	8.588	1.16E-16	1.33E-15	26.981
LTK	1.234	8.532	1.79E-16	2.00E-15	26.559
MYH7B	1.255	8.528	1.83E-16	2.05E-15	26.534
SULT1A3	1.172	8.514	2.04E-16	2.28E-15	26.427
BEST4	1.071	8.505	2.19E-16	2.43E-15	26.358
NPW	1.043	8.438	3.60E-16	3.94E-15	25.87

TMEM89	1.246	8.41	4.44E-16	4.83E-15	25.663
ANO9	1.052	8.336	7.71E-16	8.23E-15	25.12
LHB	1.258	8.305	9.70E-16	1.02E-14	24.895
ANKK1	1.037	8.285	1.12E-15	1.17E-14	24.751
CATSPER4	1.308	8.282	1.15E-15	1.20E-14	24.727
COX6B2	1.222	8.268	1.28E-15	1.32E-14	24.625
PLGLB1	1.043	8.228	1.71E-15	1.74E-14	24.337
DNAJB13	1.107	8.187	2.30E-15	2.31E-14	24.046
SGK2	1.317	8.179	2.44E-15	2.45E-14	23.988
FKBP6	1.247	8.14	3.24E-15	3.19E-14	23.71
ENO3	1.021	8.128	3.54E-15	3.47E-14	23.623
FGF22	1.059	8.128	3.54E-15	3.47E-14	23.622
AMN	1.336	8.125	3.61E-15	3.53E-14	23.602
TMC2	1.207	8.091	4.66E-15	4.49E-14	23.353
VGF	1.468	8.087	4.79E-15	4.60E-14	23.326
NR2E3	1.05	8.074	5.24E-15	5.02E-14	23.238
RUFY4	1.535	8.054	6.06E-15	5.76E-14	23.095
POU5F1	1.114	8.043	6.58E-15	6.23E-14	23.013
ATP2A1	1.002	7.989	9.70E-15	8.96E-14	22.632
JSRP1	1.189	7.948	1.30E-14	1.19E-13	22.342
CGB7	1.435	7.919	1.60E-14	1.45E-13	22.139
IZUMO1	1.22	7.911	1.69E-14	1.53E-13	22.086
PRAP1	1.239	7.884	2.05E-14	1.84E-13	21.896
MSLNL	1.404	7.872	2.23E-14	1.98E-13	21.814
AMH	1.878	7.866	2.34E-14	2.07E-13	21.769
ATP6V1B1	1.344	7.856	2.51E-14	2.21E-13	21.7
CLDN9	1.297	7.819	3.24E-14	2.83E-13	21.448
UPK3B	1.066	7.818	3.27E-14	2.85E-13	21.439
CTRC	1.258	7.781	4.25E-14	3.66E-13	21.183
PAGE2B	1.144	7.755	5.11E-14	4.37E-13	21.001
ALDH8A1	1.017	7.747	5.41E-14	4.60E-13	20.945
SLC22A14	1.021	7.734	5.91E-14	5.00E-13	20.859
TRIM17	1.104	7.683	8.47E-14	7.08E-13	20.505
CPNE7	1.354	7.67	9.26E-14	7.69E-13	20.419
ZAP70	1.052	7.639	1.15E-13	9.45E-13	20.207
IFITM5	1.091	7.592	1.59E-13	1.29E-12	19.886
UCN2	1.057	7.555	2.05E-13	1.64E-12	19.639
RAPSN	1.357	7.521	2.59E-13	2.05E-12	19.409
VSIG8	1.174	7.515	2.69E-13	2.12E-12	19.371
CRYBA2	1.054	7.332	9.35E-13	6.94E-12	18.152
KLKB1	1.087	7.263	1.48E-12	1.08E-11	17.701
CRYBB2	1.102	7.254	1.58E-12	1.15E-11	17.638
OXT	1.087	7.205	2.19E-12	1.57E-11	17.321
TBC1D3B	1.098	7.188	2.44E-12	1.75E-11	17.211

VWA3A	1.15	7.15	3.16E-12	2.23E-11	16.961
AANAT	1.037	7.147	3.21E-12	2.26E-11	16.944
EML5	-1.1	-7.14	3.26E-12	2.29E-11	16.929
NKAIN4	1.211	7.11	4.10E-12	2.85E-11	16.706
RHOXF1	1.299	7.091	4.64E-12	3.21E-11	16.584
ST8SIA6	-1.26	-7.04	6.31E-12	4.29E-11	16.283
TTC34	1.051	7.003	8.22E-12	5.52E-11	16.025
KRTAP5-8	1.037	6.965	1.06E-11	7.03E-11	15.779
CEL	1.12	6.795	3.13E-11	1.97E-10	14.719
GNG8	1.006	6.724	4.90E-11	3.02E-10	14.28
CCDC114	1.086	6.698	5.78E-11	3.53E-10	14.119
CLEC18C	1.147	6.695	5.89E-11	3.59E-10	14.1
AFF3	-1.08	-6.65	7.59E-11	4.58E-10	13.853
OR2H2	1.05	6.625	9.10E-11	5.43E-10	13.675
LCN10	1.288	6.619	9.43E-11	5.62E-10	13.641
DEFB124	1	6.599	1.07E-10	6.32E-10	13.52
MMP23B	1.082	6.582	1.19E-10	7.02E-10	13.414
TCTEX1D4	1.147	6.56	1.36E-10	7.96E-10	13.284
CYP4Z1	1.142	6.504	1.93E-10	1.11E-09	12.944
SLC9A3	1.19	6.502	1.94E-10	1.12E-09	12.937
SCRT1	1.016	6.469	2.38E-10	1.36E-09	12.737
CAPN8	1.188	6.404	3.54E-10	1.98E-09	12.351
TBC1D3E	1.259	6.392	3.80E-10	2.12E-09	12.281
ENTPD8	1.048	6.39	3.84E-10	2.14E-09	12.273
AKR1C4	1.048	6.39	3.86E-10	2.15E-09	12.268
DLGAP1	-1.2	-6.37	4.33E-10	2.40E-09	12.155
KIF12	1.065	6.318	5.92E-10	3.23E-09	11.851
NEU4	1.127	6.306	6.38E-10	3.46E-09	11.778
LENEP	1.016	6.284	7.26E-10	3.90E-09	11.652
RSPO4	1.039	6.255	8.62E-10	4.61E-09	11.485
KISS1	1.035	6.241	9.36E-10	4.98E-09	11.405
GABRE	1.151	6.191	1.26E-09	6.56E-09	11.115
SYT8	1.411	6.159	1.52E-09	7.85E-09	10.93
KRTDAP	1.069	6.145	1.65E-09	8.47E-09	10.851
TNNI3	1.436	6.048	2.90E-09	1.45E-08	10.305
FCGR3B	-1.29	-6.04	3.01E-09	1.50E-08	10.267
IFNE	1.114	6.012	3.57E-09	1.77E-08	10.101
TERT	1.079	5.983	4.21E-09	2.06E-08	9.9427
KRT72	1.429	5.949	5.12E-09	2.49E-08	9.7526
MYBPC3	1.134	5.936	5.51E-09	2.67E-08	9.6809
TEKT2	1.021	5.908	6.46E-09	3.10E-08	9.526
PNMA5	1.168	5.831	9.95E-09	4.67E-08	9.1066
LBX2	1.001	5.78	1.33E-08	6.13E-08	8.827
EPHA5	-1.38	-5.69	2.21E-08	9.95E-08	8.3321

SLC17A1	1.01	5.679	2.32E-08	1.04E-07	8.2852
NKX6-3	1.102	5.61	3.38E-08	1.49E-07	7.9214
LCNL1	1.078	5.587	3.83E-08	1.68E-07	7.7999
PCSK1N	1.065	5.479	6.84E-08	2.91E-07	7.2396
SULT1E1	1.174	5.439	8.45E-08	3.54E-07	7.0358
ABCC12	1.529	5.428	8.93E-08	3.73E-07	6.9819
CXCR1	-1.12	-5.43	9.00E-08	3.75E-07	6.9749
LY6G6C	1.06	5.38	1.16E-07	4.76E-07	6.7328
LUZP2	-1.01	-5.37	1.22E-07	5.00E-07	6.6807
ZSCAN1	1.165	5.361	1.27E-07	5.19E-07	6.6411
KISS1R	1.059	5.337	1.44E-07	5.86E-07	6.5183
BTBD16	1.016	5.313	1.63E-07	6.58E-07	6.3988
CYP2W1	1.055	5.212	2.76E-07	1.08E-06	5.8945
KRT81	1.158	5.145	3.86E-07	1.48E-06	5.5689
SLC6A14	-1.22	-5.02	7.25E-07	2.67E-06	4.9629
TMEM190	1.026	5.007	7.69E-07	2.82E-06	4.906
OR51E2	-1.21	-4.64	4.44E-06	1.44E-05	3.2251
BARX1	1.02	4.333	1.78E-05	5.28E-05	1.8977
UCN3	1.017	4.189	3.32E-05	9.39E-05	1.3073
SLC17A4	1.365	4.058	5.76E-05	0.00015679	0.7855
ALB	1.052	3.995	7.45E-05	0.00019819	0.5434
SLC26A3	-1.05	-3.69	0.000247	0.00060349	-0.583
RPE65	-1.08	-3.64	0.000307	0.00073766	-0.786
SULT4A1	1.028	3.538	0.000441	0.0010357	-1.126
SERPINB11	1.334	3.485	0.000537	0.00124413	-1.309
APOH	1.132	3.342	0.000896	0.00199401	-1.784
UNC5D	-1.02	-3.12	0.001898	0.00395987	-2.476
ALOX15B	-1.06	-3.01	0.00277	0.00559724	-2.821
GC	1.048	2.916	0.003712	0.00731106	-3.088
ORM1	-1.23	-2.43	0.015319	0.02652601	-4.356

Table S6. The 21-gene signature and formula for calculating the HIS_score.

No.	Gene	Risk Direction
1	MXD3	Positive
2	CCDC28B	Positive
3	COL11A2	Positive
4	SLC39A5	Positive
5	GPT	Positive
6	DNASE1L2	Positive
7	PIF1	Positive

8	KRTAP5-9	Positive
9	TTLL10	Positive
10	KRTAP5-1	Positive
11	KRTAP5-10	Positive
12	HAGHL	Positive
13	MSLNL	Positive
14	AMH	Positive
15	NKAIN4	Positive
16	CCDC114	Positive
17	SLC9A3	Positive
18	SULT1E1	Positive
19	ALB	Positive
20	SLC6A14	Negative
21	RPE65	Negative

The HIS_score was calculated as follows: HIS_score = mean expression of the 19 positive genes − mean expression of the 2 negative genes, where positive genes are defined as those whose high expression is associated with higher HIS_score and worse prognosis, and negative genes are defined as those whose high expression is associated with lower HIS_score and better prognosis.

Supplementary Figures:

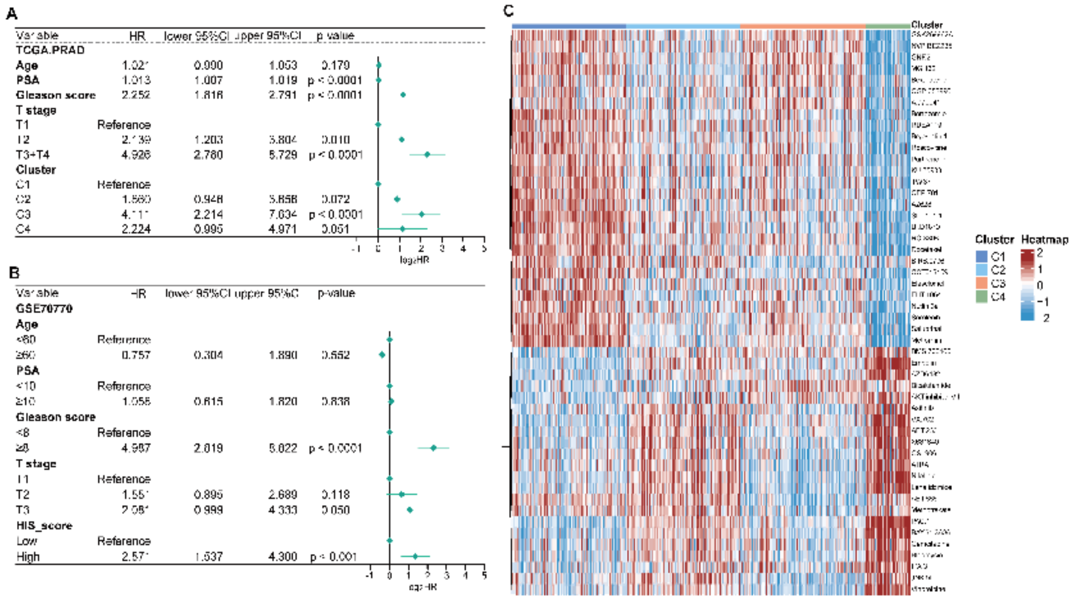

Figure S1. Differences in hazard ratios and drug sensitivities across the PRAD clusters. (**A, B**) Forest plots illustrating the impacts of the four clusters alongside clinicopathological characteristics (age, PSA, Gleason score, and T stage) on PFI (**A**) and DFI (**B**), as determined by univariate analysis. (**C**) Heatmap demonstrating the differences in IC50 values of 50 chemotherapeutic drugs (P < 0.05) from the GDSC database across the four PRAD clusters.

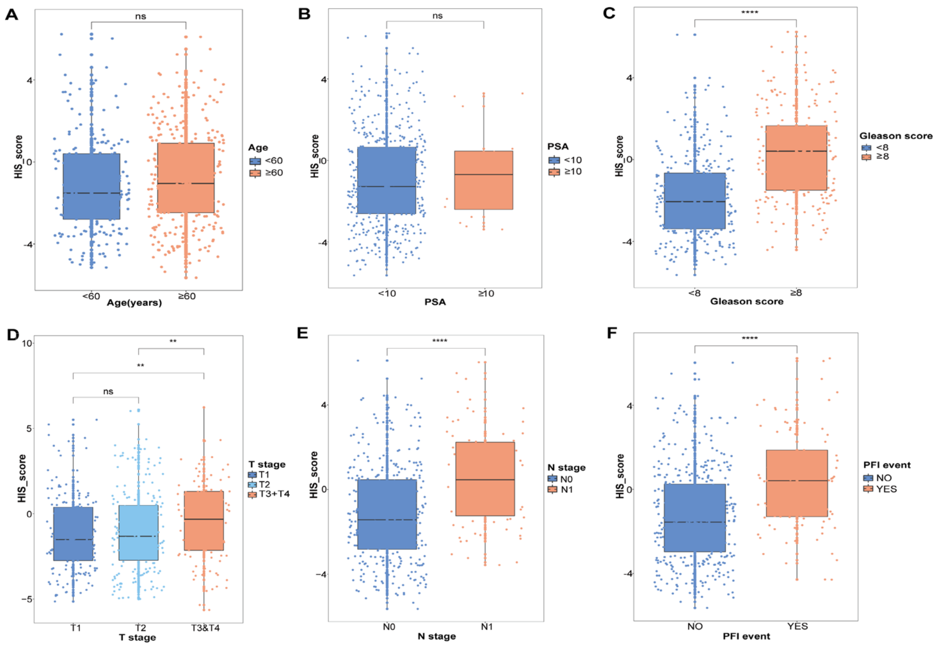

Figure S2. Associations between the HIS_score and clinical characteristics of PCa patients. (**A–F**) Distribution of HIS_score values for patients stratified by different clinical characteristics in the TCGA-PRAD cohort. These characteristics include age (**A**), PSA (**B**), Gleason score (**C**), T stage (**D**), N stage (**E**), and Event (**F**) (Event: “YES” indicates the occurrence of BCR, clinical progression (local or distant), or death from any cause during follow-up; “NO” indicates patients who were censored at the last follow-up without an event).

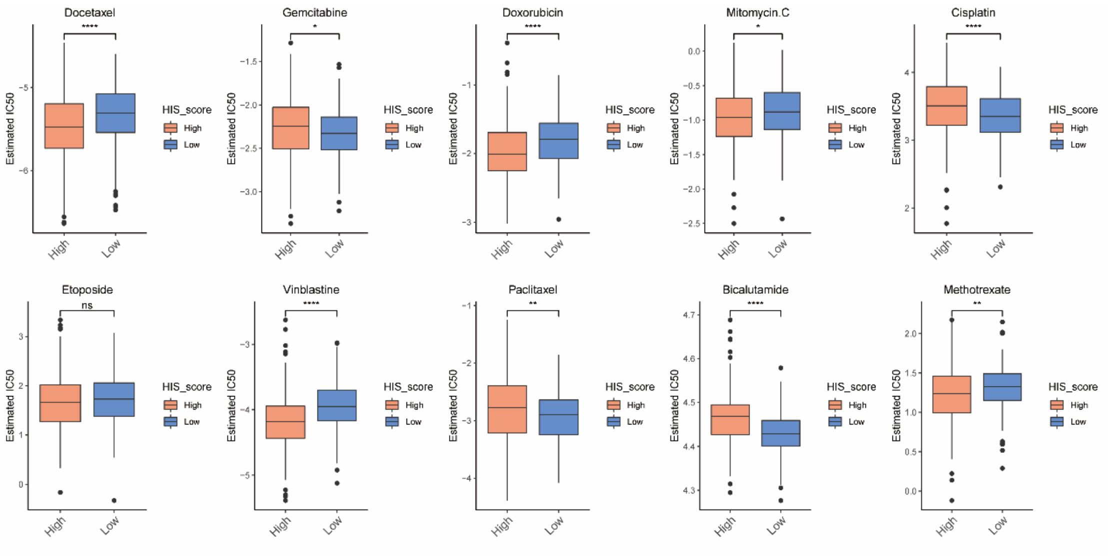

Figure S3. Differences in drug sensitivity between the high and low HIS_score subgroups. Comparison of the IC50 values for 10 common chemotherapeutic drugs between the high and low HIS_score subgroups in the TCGA-PRAD cohort.
