# Supplementary figures and images for: A Gene Feature Based on Histone Modifications Can Predict the Prognosis of Prostate Cancer
Source: Biomedicines. 2026 May 28;14(6):1219. doi: 10.3390/biomedicines14061219 (PMC13296647; doi:10.3390/biomedicines14061219)

**A**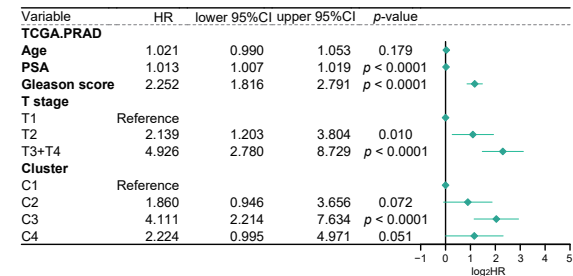**B**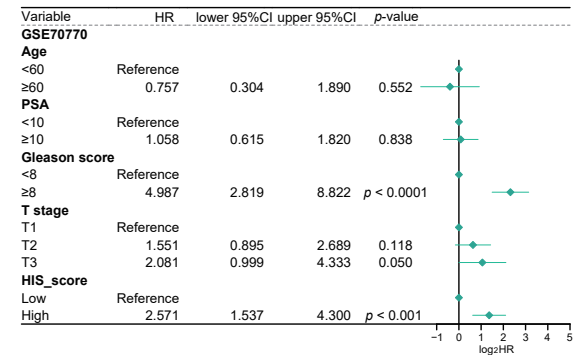**C**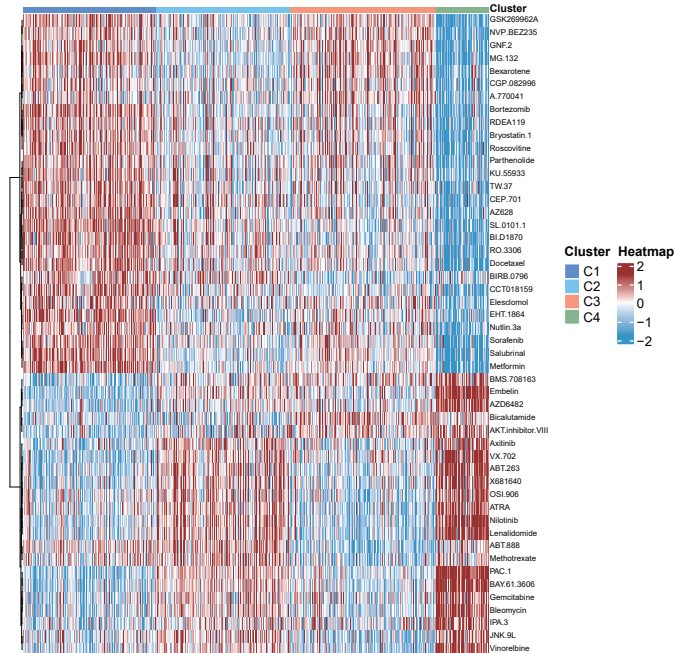

Supplement: Supplementary file 1 [file biomedicines-14-01219-s001.zip › biomedicines-4179553-supplementary/Figure S1.pdf]

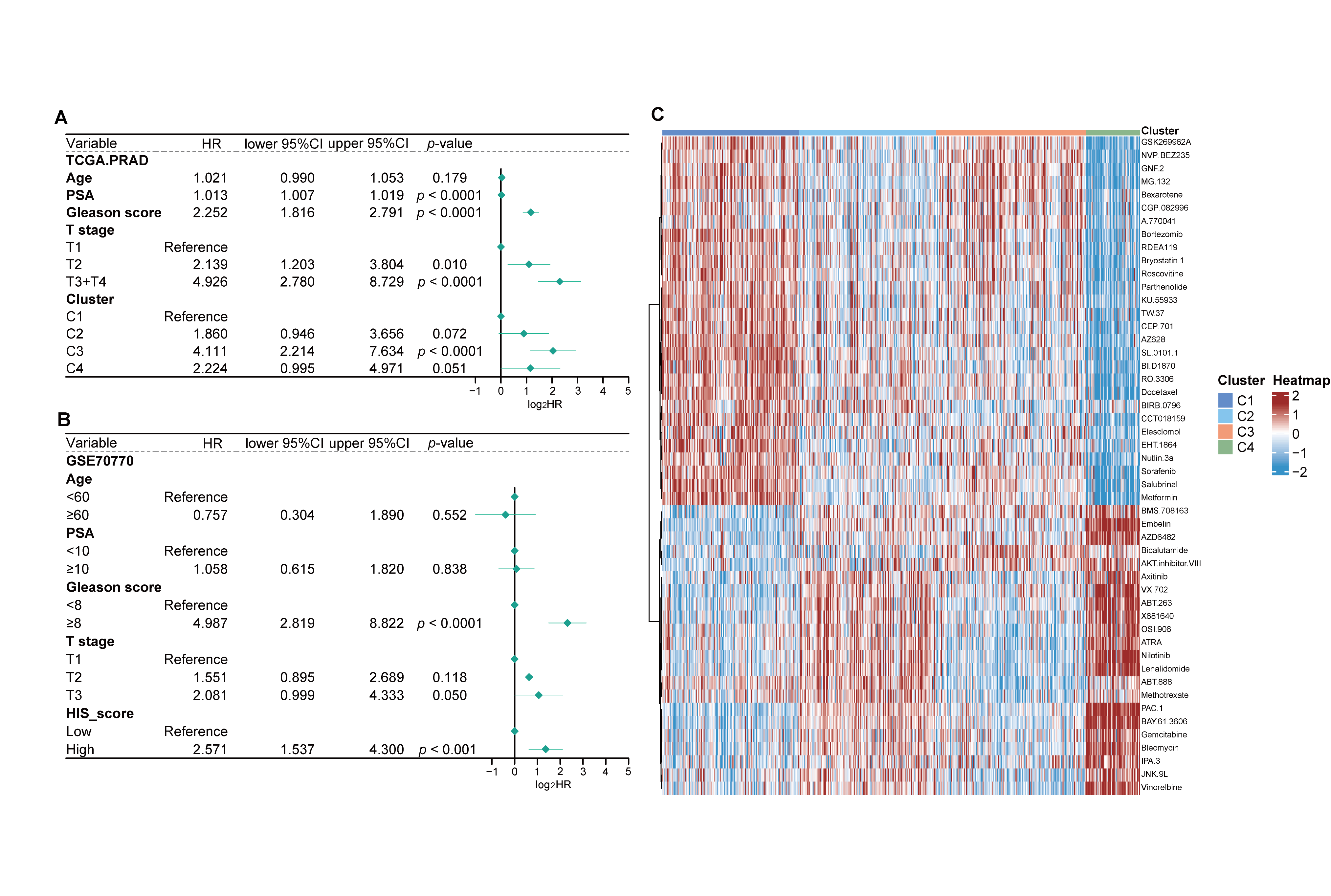

Supplement: Supplementary file 1 [file biomedicines-14-01219-s001.zip › biomedicines-4179553-supplementary/Figure S1.tif]

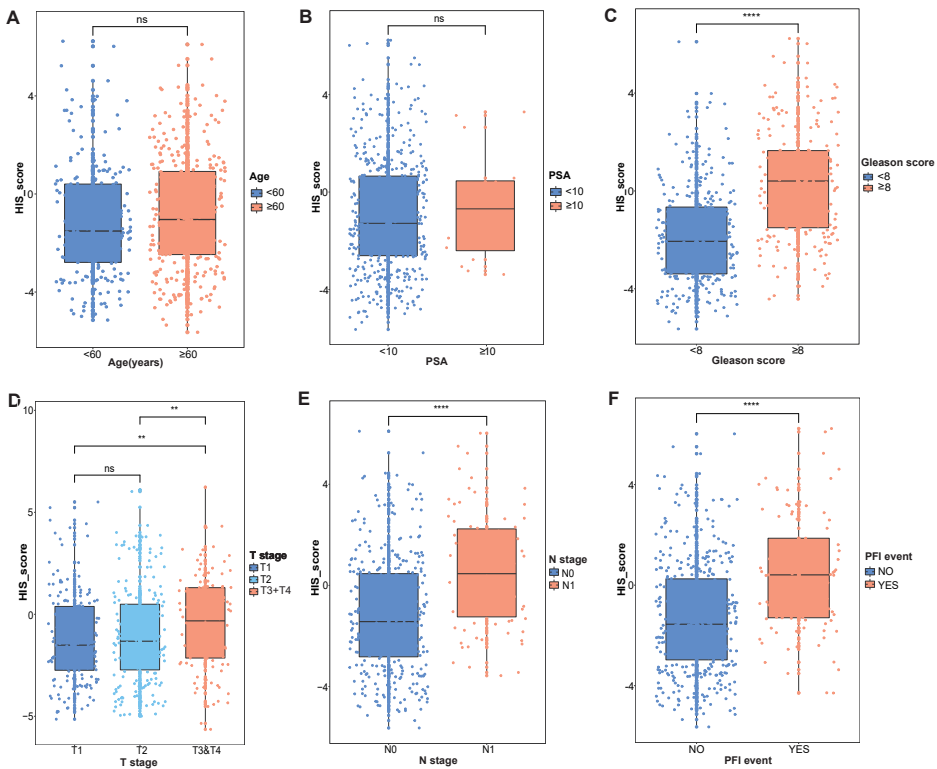

Supplement: Supplementary file 1 [file biomedicines-14-01219-s001.zip › biomedicines-4179553-supplementary/Figure S2.pdf]

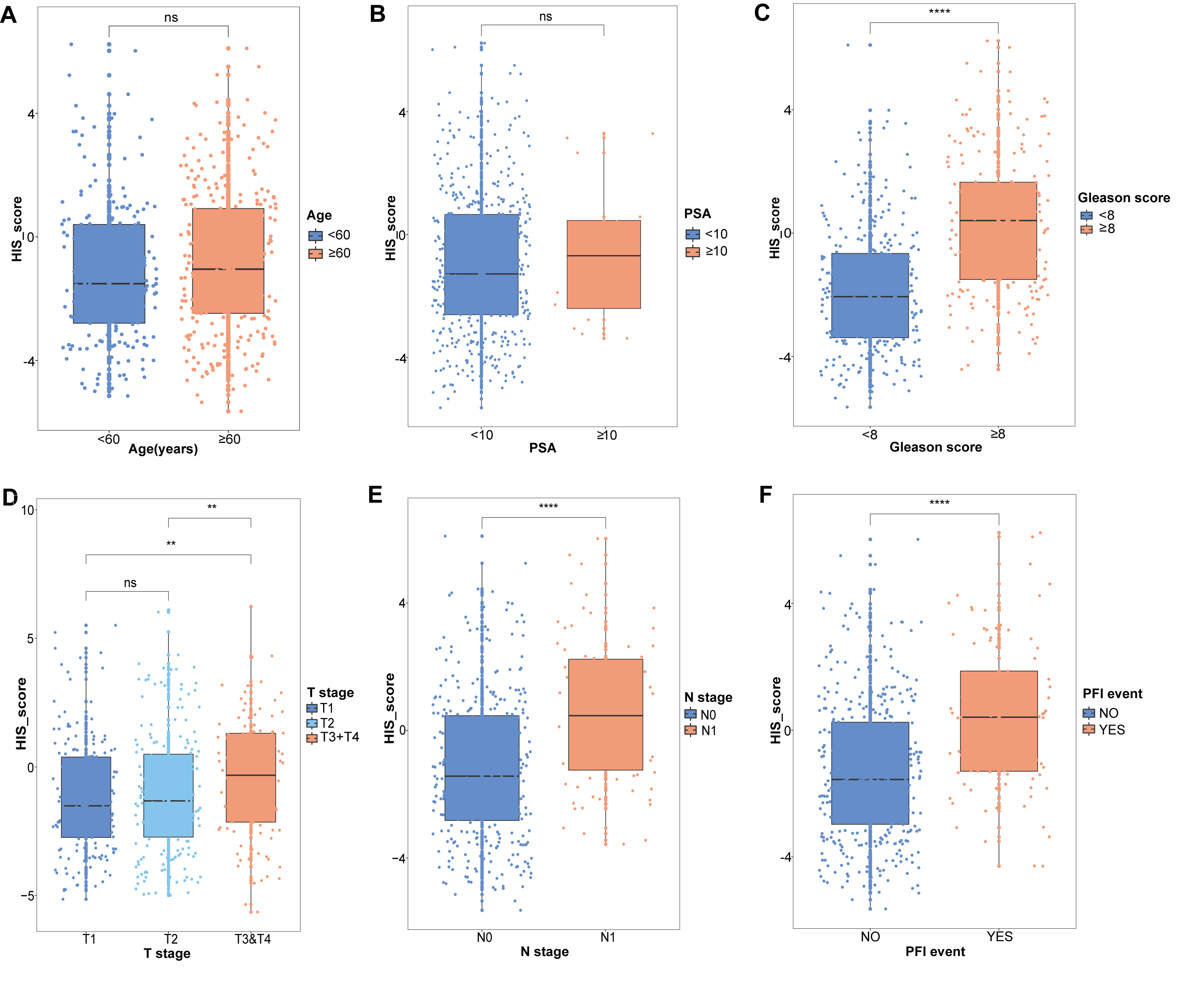

Supplement: Supplementary file 1 [file biomedicines-14-01219-s001.zip › biomedicines-4179553-supplementary/Figure S2.tif]

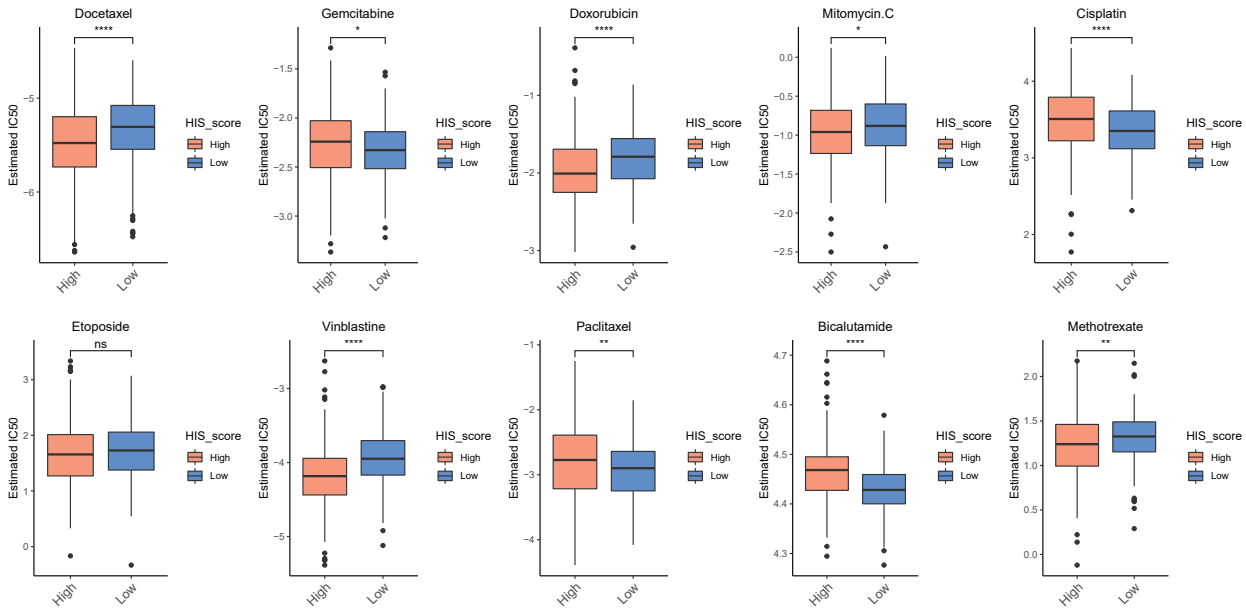

Supplement: Supplementary file 1 [file biomedicines-14-01219-s001.zip › biomedicines-4179553-supplementary/Figure S3.pdf]

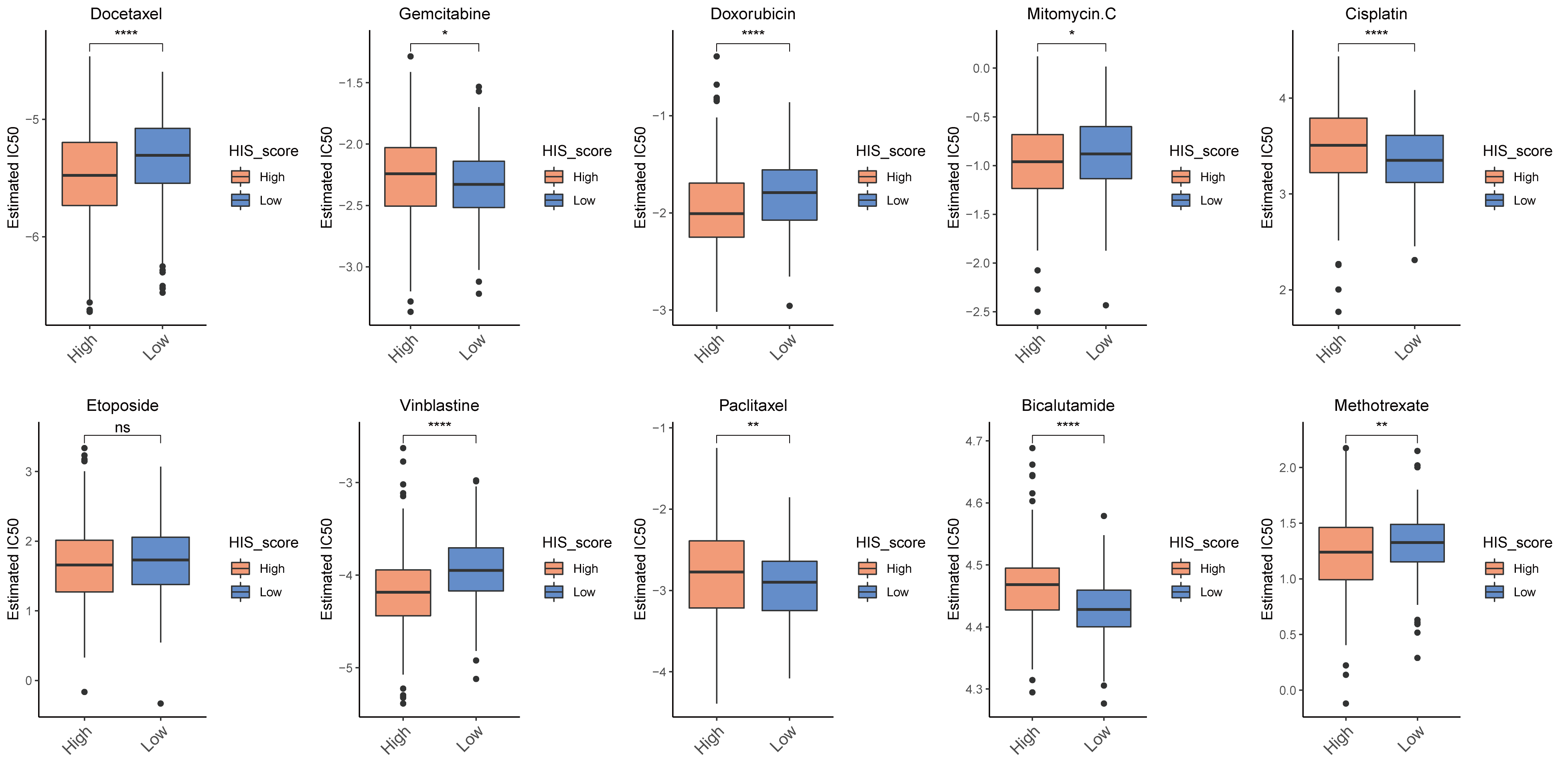

Supplement: Supplementary file 1 [file biomedicines-14-01219-s001.zip › biomedicines-4179553-supplementary/Figure S3.tif]
